# TMT-Based Proteomic Analysis Reveals the Molecular Mechanisms of Sodium Pheophorbide A against Black Spot Needle Blight Caused by *Pestalotiopsis neglecta* in *Pinus sylvestris* var. *mongolica*

**DOI:** 10.3390/jof10020102

**Published:** 2024-01-26

**Authors:** Yundi Zhang, Jing Yang, Shuren Wang, Yunze Chen, Guocai Zhang

**Affiliations:** 1 Heilongjiang Province Key Laboratory of Forest Protection, School of Forest, Northeast Forestry University, Harbin 150040, China; yundi1103@126.com (Y.Z.); jingyang@gzu.edu.cn (J.Y.); wang02102022@126.com (S.W.); 2 College of Forestry, Guizhou University, Guiyang 550025, China; 3 School of Biological Sciences, Guizhou Education University, Guiyang 550018, China

**Keywords:** antifungal mechanism, sodium pheophorbide A, *Pestalotiopsis neglecta*, *Pinus sylvestris* var. *mongolica*, proteomic analysis

## Abstract

Black spot needle blight is a minor disease in Mongolian Scots pine (*Pinus sylvestris* var. *mongolica*) caused by *Pestalotiopsis neglecta*, but it can cause economic losses in severe cases. Sodium pheophorbide a (SPA), an intermediate product of the chlorophyll metabolism pathway, is a compound with photoactivated antifungal activity, which has been previously shown to inhibit the growth of *P. neglecta*. In this study, SPA significantly reduced the incidence and disease index and enhanced the chlorophyll content and antioxidant enzyme activities of *P. sylvestris* var. *mongolica*. To further study the molecular mechanism of the inhibition, we conducted a comparative proteomic analysis of *P. neglecta* mycelia with and without SPA treatment. The cellular proteins were obtained from *P. neglecta* mycelial samples and subjected to a tandem mass tag (TMT)-labelling LC-MS/MS analysis. Based on the results of de novo transcriptome assembly, 613 differentially expressed proteins (DEPs) (*p* < 0.05) were identified, of which 360 were upregulated and 253 downregulated. The 527 annotated DEPs were classified into 50 functional groups according to Gene Ontology and linked to 256 different pathways using the Kyoto Encyclopedia of Genes and Genomes database as a reference. A joint analysis of the transcriptome and proteomics results showed that the top three pathways were Amino acid metabolism, Carbohydrate metabolism, and Lipid metabolism. These results provide new viewpoints into the molecular mechanism of the inhibition of *P. neglecta* by SPA at the protein level and a theoretical basis for evaluating SPA as an antifungal agent to protect forests.

## 1. Introduction Species of Tree

Mongolian Scots pine (*Pinus sylvestris* var. *mongolica*), an evergreen coniferous tree, is the main and fast-growing species of tree in northeast China [1]. It not only plays a great greening and protective role in soil and water conservation, but it also has a relatively wide range of uses in industrial production. Mongolian Scots pine is the preferred material for long-lasting furniture due to its excellent material, straight texture, strong anti-degradative ability, and easy storage. Additionally, *P. sylvestris* var. *mongolica* is rich in several raw material compounds such as rosin and turpentine extracted from the trunk resin and quebracho extracted from the bark, making it a valuable industrial resource for construction, electric poles, ships, appliances, and the wood industry [2,3]. Therefore, the afforestation area is expanding, but it cannot be ignored that the occasional occurrence of diseases will lead to a low efficiency of cultivation and rate of utilization of *P. sylvestris* var. *mongolica. Pestalotiopsis* is an important fungal pathogen that causes reduced production and serious economic losses in industrial and horticultural crops. These crops include blueberry (*Vaccinium* sect. *Cyanococcus*), strawberry (*Fragaria* x *ananassa*), grape (*Vitis vinifera*), apple (*Malus domestica*), coconut (*Cocus nucifera*), rambutan (*Nephelium lappaceaum*), tea (*Camellia sinensis*), lotus mist (*Szygium samaranangense*), and mango (*Mangifera indica*), among others [4,5,6,7,8,9]. *P. clavispora* and *P. neglecta* have been identified for the first time in Chile in association with blueberry canker and twig blight [10], and *P. trachicarpicola* was reported as a novel pathogen that is the causal agent of twig blight in *Pinus bungeana* in China [11]. Chen et al. [12] reported that black spot blight in *P. sylvestris* var. *mongolica* occurs in northeast China, and it is caused by *P. neglecta* and mainly controlled by chemical fungicides. However, the misuse of synthetic chemical fungicides negatively impacts human and environmental health and may result in fungi that are resistant to fungicides [13]. These toxic effects have accelerated the efforts to develop new, less toxic, and highly effective antifungal agents.

Tetrapyrrole macrocyclic compounds are the degradation products of chlorophyll a with a specific carbon frame structure [14,15]. In addition to photosynthesis, these compounds can promote hematopoiesis and have anti-tumor, antimicrobial, antimutagenic, antiulcer, and hepatoprotective activities. Thus, tetrapyrrole macrocyclic compounds are important for synthesizing new photodynamic therapy drugs [16,17]. Pheophorbide a is the last product in the chlorophyll degradation pathway, which absorbs light at a wavelength above 670 nm and confers the green coloration of plants [18,19]. It penetrates tissues through radiation and produces singlet oxygen, hydroxyl radicals, and superoxide anions, which have pharmacological effects, such as cytotoxicity and antibacterial, anti-inflammatory, antiviral, and antioxidant activities; however, it is insoluble in water [20,21,22]. Sodium pheophorbide a (SPA) is a water-soluble sodium salt of pheophorbide a that is easy to use. This compound can effectively inhibit the growth of fungal pathogens of plant hosts, including the fungal diseases of economically important horticultural crops, such as cherry tomato gray mold caused by gray mold (*Botrytis cinerea*) [23]. SPA exerts antifungal activities by altering the hyphal morphology, reducing the cell wall integrity, increasing the permeability of the cell membrane, and increasing the activity of defense system-related enzymes in cherry tomatoes.

The latest advances in high-throughput omics technology, including transcriptomics and proteomics, among others, provide a new way to study the growth, virulence, and pathogenic mechanism of plant-pathogenic fungi. Typically, antifungal agents for agricultural and forestry cash crops primarily act by destroying the stability of cell membranes and inhibiting the biosynthesis of proteins and nucleic acids and cell respiration. Shi et al. [24] revealed the mechanisms of *Botrytis cinerea* controlled with the nucleoside antibiotic wuyiencin using an iTRAQ-based proteomic analysis. Based on a comparative proteomic analysis of two-dimensional (2D) gel electrophoresis, 21 differentially expressed proteins (DEPs) were identified in *B. cinerea* spores in response to oligochitosan, and the proteomic information combined with a biochemical analysis provided the possible mechanism by which oligochitosan inhibits fungal pathogens [25]. Transcriptomic and proteomic analyses indicated that linalool downregulated the metabolic biosynthetic pathways at the transcript and protein levels to inhibit the growth of *Fusarium oxysporum* [26]. Our previous studies confirmed that SPA exhibits in vitro photoactivated antifungal activity against *P. neglecta* and explained the possible antifungal mechanism of SPA based on physiology, biochemistry, light activation, and transcription level [27]. In this study, we determined the ability of SPA to control black spot needle blight of *P. sylvestris* var. *mongolica*. The DEPs were determined using a tandem mass tag (TMT)-labelled quantitative proteome sequencing technology based on the transcriptome sequencing results. A possible mode of action of SPA on *P. neglecta* mycelium was identified in the joint analysis, which further elucidated the inhibitory effect of SPA on *P. neglecta*.

## 2. Materials and Methods

### 2.1. Reagents

Sodium pheophorbide a (SPA, 98%, average molecular weight 614.7 g/mol) was purchased from Haining Fengming Chlorophyll Co., Ltd. (Haining, China). SPA was dissolved in sterile distilled water at a concentration of 20 mg·mL^−1^ to create a stock solution. Superoxide dismutase (SOD, Cat. No. A001-3-1), peroxidase (POD, Cat. No. A084-3-1), and catalase (CAT, Cat. No. A007-1-1) test kits were purchased from Nanjing Jiancheng Biotechnology Co., Ltd. (Nanjing, China). All the other chemicals were of analytical grade and were used without further purification.

### 2.2. Plant Materials and Pathogen Cultivation

Healthy biennial seedlings of *P. sylvestris* var. *mongolica* were provided by Yuanlin Nursery of the Kudur Forestry Bureau (longitude 120°53′–121°59′ E, latitude 49°36′–50°16′ N, Hulunbuir, Inner Mongolia, China). A total of 120 seedlings were separated into three treatment groups. The seedlings were inspected for pests and diseases before initiating the experiment, and their surfaces were then disinfected with 75% (*v*/*v*) ethanol.

*P. neglecta* was supplied by the Heilongjiang Province Key Laboratory of Forest Protection (Northeast Forestry University, Harbin, China) and was cultured on potato dextrose agar (PDA) media at 25 °C for 7 days. Thereafter, sterile water was poured onto plates containing the mycelia, followed by vortexing for 30 s and filtering through sterilized cotton to obtain spore suspensions. The spore concentrations were adjusted to 1 × 10^6^ spores mL^−1^ using a hemocytometer under 400× magnification of the inversed optical microscope (Motic B1 Series; Motic Instruments, Richmond, BC, Canada). 

### 2.3. Antifungal Activity of SPA

A volume of 5 mL of different concentrations of SPA (10 and 20 mg·mL^−1^) was sprayed on all the branches of *P. sylvestris* var. *mongolica* seedlings, and the control group was sprayed with sterile water. After natural drying, each plant was evenly sprayed with 10 mL of spore suspension. The antifungal effects were investigated 30 days after the treatment, and the needles on each seedling were randomly sampled to determine the chlorophyll content and activity of the antioxidant enzymes.

### 2.4. Protein Extraction

SPA powder was added to autoclaved liquid media (potato dextrose broth [PDB], pH 6.0), at a final concentration of 0.5 mg·mL^−1^. Each Erlenmeyer flask contained 80 mL of PDB (with 8 mL of SPA) inoculated with 0.8 mL of spore suspension. The Erlenmeyer flasks were placed in a shaking incubator and incubated for 3 days at 25 °C with a light intensity of 4000 lx and at a speed of 150 rpm. An equal amount of distilled water was added to the control. At the end of incubation, the mycelia were collected by filtration, followed by centrifugation at 8000× *g* for 10 min, and the precipitate was collected and rapidly frozen in liquid nitrogen.

A total of 0.1 g of each *P. neglecta* mycelial sample from the control and SPA-treated groups was ground into powder in liquid nitrogen and mixed with 0.5 mL of TCA/acetone (1:9 *v*/*v*) by vortexing. The mixture was then precipitated at −20 °C for more than 4 h, after which the solution was centrifuged at 6000× *g* for 40 min at 4 °C, and the supernatant was discarded. Pre-cooled acetone solution was added to the precipitate, and the precipitate was washed three times and dried in a fume hood.

SDT lysate (600 μL) was added to 20 mg of the dried powder to resuspend the precipitate, and the suspension was vortexed and boiled in a water bath for 5 min. After ultrasonic crushing, the solution was incubated in a boiling water bath for 15 min. The solution was then centrifuged at 14,000× *g* for 15 min, and the supernatant was collected by filtration using a sterile 0.22 μm filter. The bicinchoninic acid (BCA) method was used to quantify the protein, and the rest of the samples were stored at −20 °C.

### 2.5. FASP Digestion of Proteins

Dithiothreitol (DTT) was added to 200 μg of protein per sample to a final concentration of 100 mM, boiled for 5 min, and cooled to room temperature. Thereafter, 200 μL of urea buffer (UA buffer; 8 M urea, 150 mM Tris-HCl, pH 8.5) was mixed with the samples and transferred to a 30 kD ultrafiltration centrifuge tube. The mixture was centrifuged at 12,500× *g* for 25 min, and the filtrate was discarded. This step was repeated twice. After adding 100 μL of iodoacetamide buffer (IAA buffer containing 100 mM of IAA in UA) to the precipitate, the mixture was vortexed at 600 rpm for 1 min, incubated at room temperature for 30 min, and then centrifuged at 12,500× *g* for 25 min. A volume of 100 μL of UA buffer was added again to the mixture, followed by centrifugation at 12,500× *g* for 15 min, and the process was repeated twice. Thereafter, 100 μL of 0.1 M tetraethylammonium bromide (TEAB) solution was added, and the mixture was centrifuged at 12,500× *g* for 15 min. This procedure was repeated twice. A volume of 40 μL of trypsin buffer (4 μg trypsin in 40 μL 0.1 M TEAB solution) was then added to the sample and mixed by vortexing at 600 rpm for 1 min, followed by incubation at 37 °C for 16–18 h. A new collection tube was used to centrifuge the samples at 12,500× *g* for 15 min. Finally, 20 μL of 0.1 M TEAB solution was added, and the solution was centrifuged at 12,500× *g* for 15 min, after which the filtrate was collected.

### 2.6. TMT Labeling

A total of 100 μg of peptides were taken from each sample and labeled using the TMT Labeling Kit according to the manufacturer’s instructions (Thermo Scientific TMT Labeling Kit, Thermo Fisher Scientific, Waltham, MA, USA). Each set of labeled peptides was mixed and fractionated using an Agilent 1260 infinity II HPLC system (Agilent Technologies, Santa Clara, CA, USA). Buffer A contained 10 mM ammonium formate and 5% acetonitrile, with a pH of 10.0, while Buffer B contained 10 mM ammonium formate and 85% acetonitrile, with a pH of 10.0. The chromatographic column was equilibrated with Buffer A, and the sample was loaded from the autosampler to the column for separation at a flow rate of 1 mL min^−1^. The liquid-phase gradient was as follows: 0–25 min: Buffer B 0%; 25–30 min: linear gradient of Buffer B from 0–7%; 30–65 min: linear gradient of Buffer B from 7−40%; 65−70 min: linear gradient of Buffer B from 40−100%; and 70 min to 85 min: Buffer B was maintained at 100%. The absorbance was monitored at 214 nm during the elution process, and the eluted fractions were collected every 1 min. Approximately 40 fractions were collected, and the samples were lyophilized and reconstituted with 0.1% formic acid (FA) to N parts.

### 2.7. EASY nLC and Mass Spectrometry

Each sample was separated using a nanoliter flow rate EASY nLC system (Thermo Fisher Scientific). Buffer A was an aqueous solution of 0.1% formic acid, and B was an aqueous solution of 0.1% formic acid in an aqueous solution of 80% acetonitrile. The column was equilibrated with 100% Buffer A. The sample was loaded from an autosampler onto an analytical column (Acclaim PepMap RSLC 50 μm × 15 cm, nano viper, P/N164943; Thermo Fisher Scientific) at a flow rate of 300 nL min^−1^. The gradient was as follows: 0−3 min: Buffer B 6%; 3–45 min: Buffer B linear gradient from 6–28%; 45–50 min: Buffer B linear gradient from 28–38%; and Buffer B was maintained at 100% from 55–60 min.

After chromatography, the samples were analyzed with a Q Exactive Plus mass spectrometer (Thermo Fisher Scientific). The analysis time was 60 min and the detection method was the positive ion. The scanning range of the precursor ion was 350–1800 *m*/*z*, and the resolution of the first-order mass spectrometer was 70,000. The AGC target was 3e^6^, and the first-level Maximum IT was 50 ms. The mass-to-charge ratios of peptides and peptide fragments were collected using 10 MS2 scans generated after each full scan. The MS2 activation type was HCD, with an isolation window of 2 *m*/*z*, a secondary MS resolution of 35,000, the microscans set to 1, a secondary maximum IT of 45 ms, and a normalized collision energy of 30 eV.

### 2.8. Bioinformatic Analysis

#### 2.8.1. Protein Clustering

The quantitative information of the target set of proteins in the six samples of *P. neglecta* mycelia was first normalized. The samples and expression of the proteins were set as two dimensions, and the Euclidean algorithm was chosen to classify them using the “Average linkage” connection method in matplotlib software (version 3.1.1) to generate a hierarchical clustering heat map.

#### 2.8.2. Gene Ontology (GO) Functional Annotations

Blast2GO [28] was used to perform the GO functional annotation of the target protein set in the six samples of *P. neglecta* mycelia. After the initial functional annotation, the conserved motifs that matched the target proteins in the EBI database were searched using InterProScan [29]. The functional information associated with the conserved motifs was re-annotated to the target protein sequences to complement the previously annotated information and to establish a linkage between the different GO categories.

#### 2.8.3. KEGG Pathway Annotations

KOALA (KEGG Orthology and Links Annotation, https://www.kegg.jp/blastkoala/, accessed on 24 January 2024) [30] was used to perform KEGG pathway annotation of the target protein collections in six samples of *P. neglecta* mycelia and to obtain information on the pathways involved in the target protein sequences according to their KO categorization.

#### 2.8.4. Enrichment Analysis of the GO Annotations and KEGG Annotations

The enrichment analysis of the GO and KEGG pathway annotations was conducted on the target protein collection of the six samples of *P. neglecta* mycelia using Fisher’s exact test. The significance level of protein enrichment in a GO term or KEGG pathway was evaluated by comparing the distribution of each GO term or KEGG pathway in the target protein collection and the overall protein collection.

### 2.9. Joint Transcriptomics and Proteomics Analysis

Transcriptome data were analyzed in our previous study [27]. The association of the transcriptome and protein data was evaluated by conducting the following analyses: (1) association of the transcriptome data with the gene data; (2) association of the gene data with the protein data; (3) use of the genes as a bridge to obtain a master table of associations; (4) screening of the data by determining the significantly different data and regulatory relationships; and (5) GO and KEGG enrichment analyses. 

### 2.10. Statistical Analysis

The original data from the mass spectrometry analyses were raw files, and FDR < 0.01 was used as a screening criterion to identify and quantity the library using Mascot 2.6 and Proteome Discoverer 2.1 (Thermo Fisher Scientific) software. All the experiments were repeated three times. The mean values and standard deviations were calculated using Microsoft Excel 2019 (Redmond, WA, USA). The statistical analyses were performed using a one-way analysis of variance (ANOVA) via SPSS 24.0 (IBM, Inc., Armonk, NY, USA).

## 3. Results

### 3.1. Control Effects

**Figure 1:**
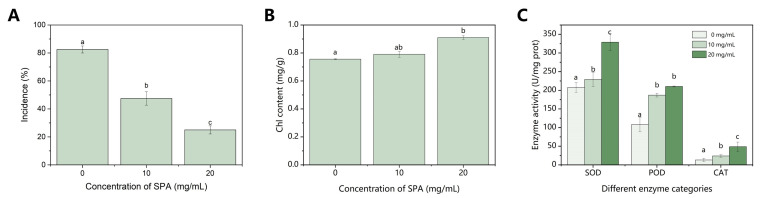
Effect of different concentrations of SPA on the incidence (**A**), chlorophyll content (**B**), and antioxidant enzyme activities (**C**) of *P. sylvestris* var. *mongolica*. SPA, sodium pheophorbide a. Bars represent the standard error of the mean (n = 3). Letters (a, b, and c) represent statistically significant differences between the treatments with different concentrations (*p* < 0.05).

The control effects, as well as the effects of SPA on the chlorophyll content and antioxidant enzyme activities in *P. sylvestris* var. *mongolica*, are shown in Figure 1. The incidence rate gradually decreased with the increasing SPA concentration, and the incidence rate of the seedlings treated with 20 mg·mL^−1^ SPA was 25% (Figure 1A). The chlorophyll content of the treatment groups was higher than that of the control group and gradually increased with increasing SPA concentration (Figure 1B). The activities of the three enzymes in the SPA-treated mycelia increased in a dose-dependent manner and were significantly higher than those of the control (Figure 1C).

### 3.2. Proteomic Evaluation of P. neglecta Treated with SPA

#### 3.2.1. Overview of the Quantitative Proteomics Analysis

**Figure 2:**
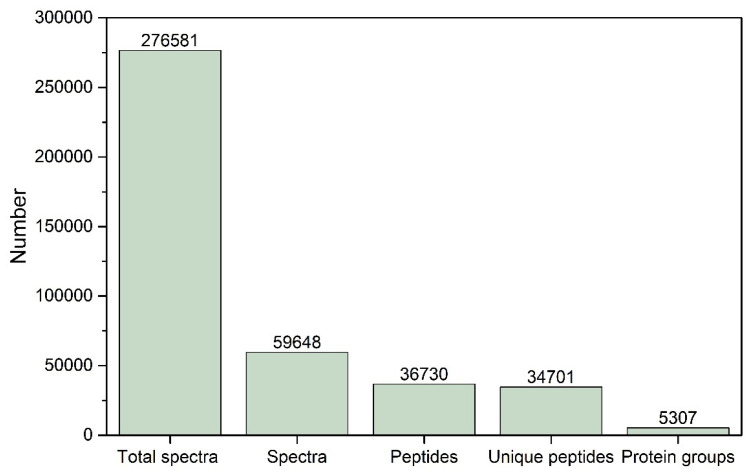
Statistics of protein identification results in TMT quantitative proteomics. TMT, tandem mass tag.

**Figure 3:**
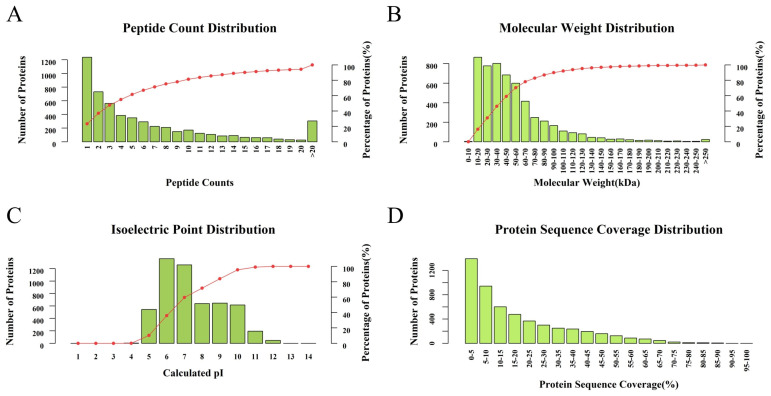
Overview of the TMT results. (**A**) Number of peptides associated with identified proteins. (**B**) Molecular weights. (**C**) Isoelectric points. (**D**) Sequence coverage for identified proteins. TMT, tandem mass tag.

A total of 276,581 spectra were generated in the TMT proteomic analysis using control *P. neglecta* and mycelia treated with SPA. As shown in Figure 2, 59,648 spectra matched known spectra and were composed of 36,730 peptides, 34,701 unique peptides, and 5307 proteins from the control *P. neglecta* and mycelia treated with SPA. As shown in Figure 3A–D, more than 78% of the proteins had at least two peptide chains. Their molecular weights ranged from 10 to 250 kDa, and their isoelectric points (pIs) ranged from 5.0 to 12.0. The proteins were mostly weakly acidic to neutral. Approximately 53% of the proteins identified had a peptide sequence coverage of more than 10%.

#### 3.2.2. Identification of the Differentially Expressed Proteins (DEPs) Using TMT

**Figure 4:**
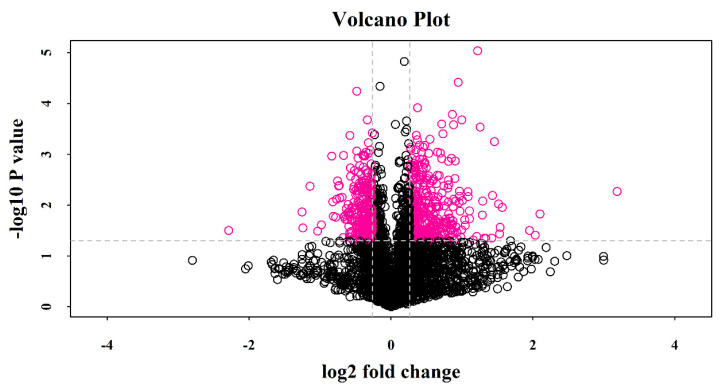
Volcano plot. Pink spots on the right represent significantly upregulated proteins, and those on the left represent downregulated ones. Black spots indicate proteins with no significant differences in their expression. Horizontal coordinate: log_2_ (fold change) indicates the logarithm of the difference in expression of multiple proteins between the two sample groups. Vertical coordinate: −log_10_ *p*-value indicates the false discovery rate obtained after correcting the significance of the *p*-value using the Benjamini–Hochberg method.

The threshold for differential expression (SPA-treated vs. control) was a 1.2-fold change in protein expression (up or down) with *p* < 0.05 (*t*-test). A total of 613 DEPs were identified in the SPA-treated sample, and 360 of them were upregulated, while 253 were downregulated (Figure 4). The details for each protein are provided in Table S1.

#### 3.2.3. Bioinformatic Analysis of the DEPs

**Figure 5:**
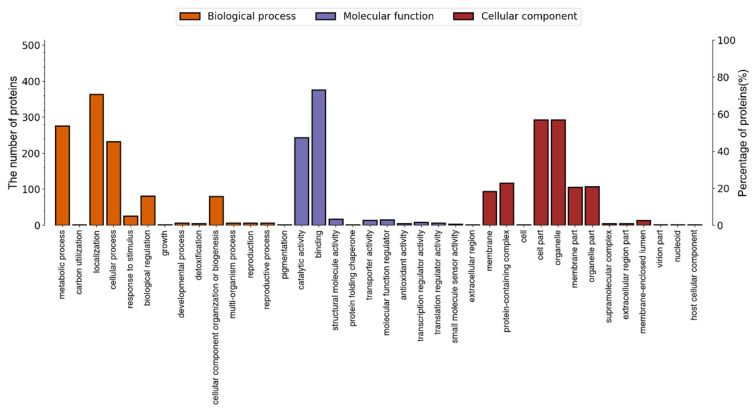
Level 2 statistics of the GO annotation. GO, Gene Ontology.

**Figure 6:**
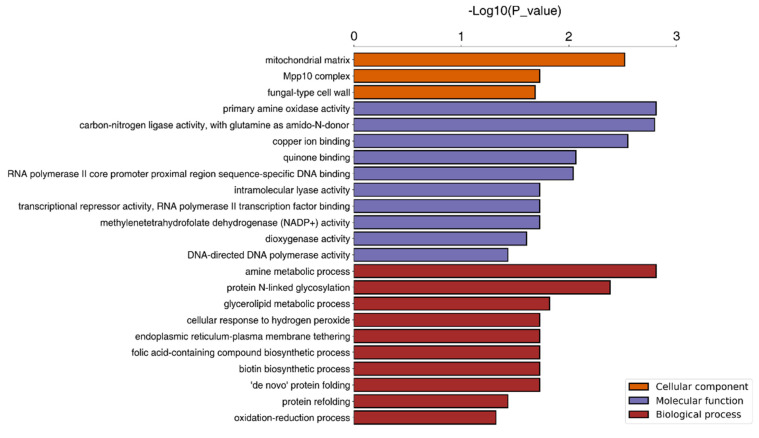
Significantly enriched GO terms. GO, Gene Ontology.

The DEPs were classified by biological process, cellular component, and molecular function. As shown in Figure 5, of the 613 DEPs, 527 were annotated and categorized into 50 functional groups. Biological processes accounted for 23 GO terms, with “cellular process” accounting for 23.61% of these and “metabolic process” accounting for 20.89%. The cellular components accounted for 14 GO terms, which were dominated by “cell part” (29.85%) and “organelle part” (17.15%). Molecular functions accounted for 13 GO terms. The most frequent were “binding” (47.03%) and “catalytic activity” (37.10%). The significance level of the enriched GO terms associated with 527 annotated proteins was assessed using Fisher’s exact test, with an adjusted *p*-value threshold of 0.05. A total of 96 significant functions were found. The top ten functions in each classification are shown in Figure 6, including “protein import into the mitochondrial matrix” (GO:0030150, *p* = 0.00046) in biological process, “chaperonin-containing T-complex” (GO:0005832, *p* = 0.00084) in cellular component, and “molecular function regulator” (GO:0098772, *p* = 0.00153) in molecular function.

**Figure 7:**
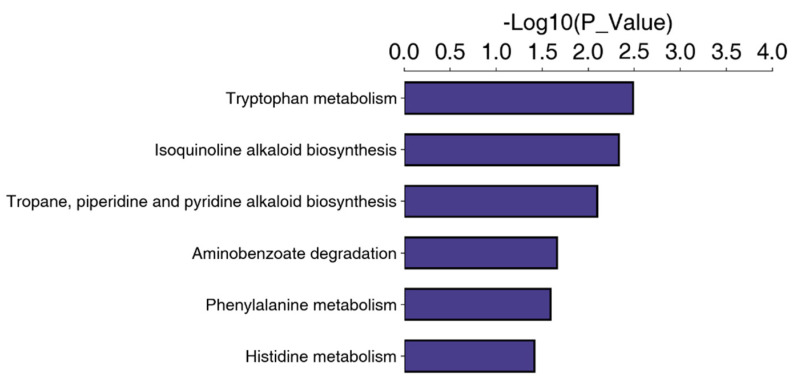
Significantly enriched KEGG pathways. KEGG, Kyoto Encyclopedia of Genes and Genomes.

Referring to the KEGG database, 527 DEPs were linked to 256 different pathways. The significance level of protein enrichment in each pathway was analyzed and calculated using Fisher’s exact test to determine which metabolic and signal transduction pathways were significantly affected. Interestingly, although many pathways were associated with DEPs, metabolism, in particular, was significantly influenced. Tryptophan metabolism, isoquinoline alkaloid biosynthesis, and tropane, piperidine, and pyridine alkaloid biosynthesis were the most significantly altered by exposure to SPA (Figure 7).

### 3.3. Joint Transcriptomics and Proteomics Analysis

**Figure 8:**
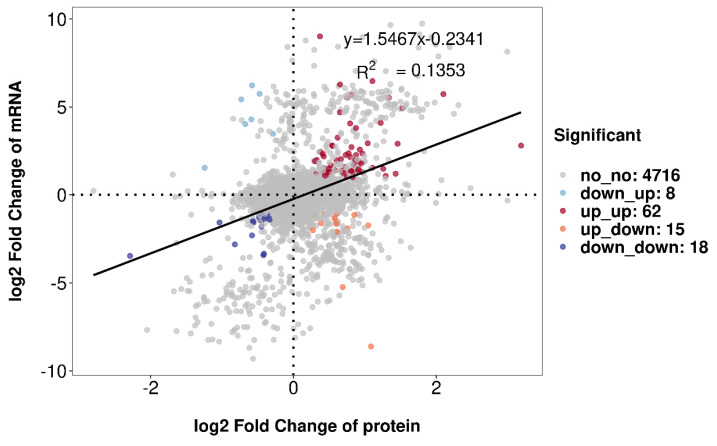
Dual-omics correlation analysis of the transcriptome and proteome. The dashed line is used to distinguish quadrants, while the solid line is the fitting equation curve for correlation analysis.

The results of a correlation analysis between the two omics are shown in Figure 8. The linear correlation equation between the transcriptomics and proteomics was y = 1.5467x − 0.2341 with a correlation coefficient of R^2^ = 0.1353. A total of 103 differentially expressed genes (DEGs) and DEPs were significantly associated between the two groups, including sixty-two “upregulated–upregulated,” fifteen “upregulated–downregulated,” eight “downregulated–upregulated,” and eighteen “downregulated–downregulated.”

**Figure 9:**
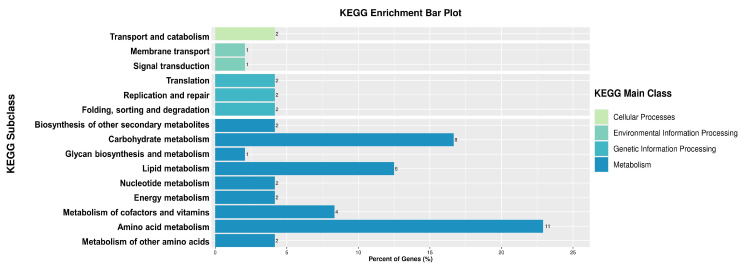
KEGG pathways associated with dual-omics. KEGG, Kyoto Encyclopedia of Genes and Genomes.

The content of the bioinformatics analysis of the proteomics data included GO functional analysis and the enrichment of differential proteins, pathway analysis, and enrichment analysis. However, in the joint analysis of the transcriptome and proteome, the focus was on pathway analysis. Genes and proteins that participated in the same pathway were screened according to the results of pathway analyses of DEPs and DEGs, and the corresponding data were comprehensively analyzed. The results are shown in Figure 9. An analysis of the integrated KEGG pathway to identify DEPs and DEGs shows the top three ranked pathways in order: Amino acid metabolism, Carbohydrate metabolism, and Lipid metabolism.

## 4. Discussion

Plant diseases caused by pathogenic fungi seriously impact the ecological security and economic efficiency of forestry and agriculture worldwide [31,32]. Although the widespread use of chemical pesticides is the primary way to control plant fungal diseases, the excessive use of chemical pesticides is harmful to the ecosystem and human health, and developing pesticides from plant sources could help reduce the negative effects caused by chemical pesticides [33,34,35]. PA, a product formed by the dephytination and demetallization of chlorophyll a in algae and higher plants, has photoactivated antifungal activity [36,37,38]. Moreover, SPA, the water-soluble sodium salt of PA, can be rapidly absorbed by plant cells and has stronger antifungal activity than PA; thus, it has the potential to be developed into a photoactivated fungicide derived from plants [39,40]. In previous studies, SPA was shown to exhibit in vitro photoactivated antifungal activity against *P. neglecta*, the causal agent of black spot needle blight of *P. sylvestris* var. *mongolica* [41]. In this study, the in vivo antifungal activity of SPA was investigated by measuring various indicators after inoculating *P. neglecta* on *P. sylvestris* var. *mongolica*. The results showed that the chlorophyll content and antioxidant enzyme activities of the seedlings increased with increasing SPA concentration. Chlorophyll is present in oxygenated photosynthetic organisms and plays a crucial role in light capture and energy transfer [42]. The increase in the chlorophyll content in *P. sylvestris* var. *mongolica* seedlings helped to improve their photosynthetic efficiency and meet their material and energy requirements.

Reactive oxygen species (ROS) are components of multiple metabolic and developmental pathways in plants under normal and stress conditions [43]. At low concentrations, ROS perform signaling functions and are involved in regulating plant growth and development and plant response to stress. However, large amounts of ROS can cause oxidative damage to lipids, nucleic acids, and proteins, eventually leading to cell death [44,45]. Under normal conditions, ROS production and scavenging are maintained in a dynamic equilibrium in plants. When plants are infected with pathogens, this equilibrium is disrupted, and the production of large amounts of ROS causes lipid peroxidation of the cell membrane, damaging the membrane systems and causing oxidative damage to plants [46]. To maintain the dynamic balance of ROS in vivo, plants have evolved a series of ROS production and scavenging mechanisms, and SOD, CAT, and POD are the key scavengers of ROS [47]. In this study, the SOD, POD, and CAT activities of the treatment group were significantly higher than those of the control group, indicating that the expression level of the antioxidant enzymes in *P. sylvestris* var. *mongolica* was increased after SPA treatment to enhance ROS scavenging. This improved the stress resistance of *P. sylvestris* var. *mongolica*. In a study of the inhibitory effect of SPA on gray mold of cherry tomato, Ji et al. [23] found that SPA significantly enhanced the activities of SOD, POD, and CAT in the cherry tomatoes to protect plant cells from oxidative damage, consistent with this study. This indicates that SPA can induce resistance to pathogenic fungi by enhancing the activities of protective enzymes in plants, thus reducing the disease incidence.

To further investigate the molecular mechanism of the inhibitory effect of SPA on *P. neglecta* at the protein level, this study utilized TMT-based quantitative proteomic sequencing to identify DEPs based on the transcriptome sequencing results. The possible modes of action of SPA against *P. neglecta* were determined via the combined dual-omics analysis. The expression levels of eight DEPs were verified with quantitative reverse transcription PCR (RT-qPCR), and the results obtained at the gene level were consistent with the proteomic analysis. This demonstrated the reliability of the proteomics results. A previous transcriptome sequencing analysis identified 3268 DEGs in *P. neglecta* treated with SPA compared to the control, including 1879 upregulated and 1389 downregulated genes. Most DEGs were involved in the metabolism of amino acids, carbohydrates, and lipids and the processing of cellular structure and genetic information [27]. In this study, a further transcriptomic analysis identified 613 DEPs, of which 360 were upregulated and 253 were downregulated. The results of a KEGG pathway enrichment analysis suggested that the DEPs were involved in tryptophan metabolism, isoquinoline alkaloid biosynthesis, and tropane, piperidine, and pyridine alkaloid biosynthesis. After the combined transcriptomic and proteomic analyses, 103 DEGs and DEPs were significantly associated between the two omics, and the KEGG metabolic pathways that were significantly affected included amino acid metabolism, carbohydrate metabolism, and lipid metabolism.

Amino acids are protein precursors involved in various metabolic pathways essential for fungal growth and development [48,49]. It has been shown that amino acids serve as a major source of nutrients for fungi, and their metabolic pathways can be used as targets for antifungal agents [50,51]. In this study, SPA upregulated some of the DEPs involved in the amino acid synthesis and metabolic pathways, such as 5-aminolevulinate synthase, NADPH-P450 reductase, and cysteine synthase, which are associated with pathways such as tryptophan metabolism; valine, leucine, and isoleucine degradation; and cysteine metabolism. Branched-chain amino acids (BCAAs) consist of leucine, isoleucine, and valine, which are essential for protein synthesis and serve as energy sources and biosynthetic precursors for cellular processes. In fungi, BCAA metabolism plays a key role in the biosynthesis of proteins, lipids, and nucleotides and the maintenance of cellular homeostasis [52,53,54]. The degradation of BCAAs is important for fungal secondary metabolism, since they are associated with the production of precursor metabolites used in secondary metabolism, such as acetyl coenzyme A (acetyl-CoA), methylmalonyl coenzyme A (MM-CoA), and propionyl coenzyme A (Pro-CoA) [55]. The downregulation of BCAA degradation can lead to the accumulation of BCAAs and reduced biosynthesis of secondary metabolites, thus affecting protein synthesis and energy metabolism in pathogenic fungi. An omics analysis showed that 2-methoxy-1,4-naphthoquinone primarily inhibits the growth of *Penicillium digitatum* by affecting the biosynthesis of BCAAs and the cell wall [56]. Thus, the changes in leucine, isoleucine, and valine in amino acid metabolism also provide insights into the antifungal targets of SPA. Furthermore, tryptophan plays an important role in osmoregulation, stomatal regulation, and ROS scavenging under stress conditions [57]. In this study, the downregulation of tryptophan metabolism after treating *P. neglecta* with SPA may have affected the biosynthetic pathways of secondary metabolites and the ability to scavenge ROS, resulting in oxidative stress in the pathogen itself. The changes in the expression level of these proteins disrupted the amino acid metabolism of the pathogenic fungi, thereby inhibiting their growth and development and reducing their viability. Thus, SPA can alter the metabolism of amino acids and protein levels in *P. neglecta*, and these changes further affect oxidative stress, the TCA cycle, energy metabolism, fatty acid biosynthesis, and other pathways in the pathogen.

Mitochondria are the primary sites of aerobic respiration in eukaryotic cells and play a central role in energetics, the TCA cycle, the ATP production pathway, and apoptosis [58]. The TCA cycle is the central pathway of energy and carbon metabolism and is the link between carbohydrate, lipid, and amino acid metabolism [59,60]. Many studies have suggested that the inhibition mechanism of antifungal agents lies in their inhibition of enzymes associated with energy metabolism in pathogenic fungi, which affects the normal function of mitochondria [61]. In this study, several proteins associated with the mitochondrial respiratory chain, including malate dehydrogenase (MDH), fumarate hydratase (FH), acetyl-CoA acetyltransferase, ATP synthase subunit epsilon, and glutamine synthetase (GS), were downregulated after treatment with SPA. The affected pathways involved the TCA cycle, 2-oxocarboxylic acid metabolism, and glutamine metabolism. MDH, an important oxidoreductase in the TCA cycle, catalyzes the dehydrogenation of L-malate to oxaloacetate, and its reduced expression restricts the biosynthesis of oxaloacetate. The active fraction of poplar (*Populus* sp.) buds inhibited the TCA cycle by reducing the activity of MDH in *Penicillium italicum* [62]. Fumarate hydratase (FH) is a catalytic enzyme that catalyzes the hydration reaction of fumaric acid into succinic acid, one of the important steps in the TCA cycle in living organisms and an important part of intracellular energy metabolism. After treatment of *B. cinerea* with tea tree oil, MDH, FH, isocitrate dehydrogenase (ICDH), acetyl-CoA acetyltransferase, homoaconitase, and dihydroxy-acid dehydratase were significantly downregulated, and this disrupted the mitochondrial respiratory chain and the TCA cycle, thus leading to cellular dysfunction and ultimately, cell death [63]. GS is considered to be the central and key enzyme in nitrogen assimilation and glutamine biosynthesis. It catalyzes the conversion of glutamate to glutamine in combination with glutamate synthase and later, in the presence of glutaminase, is converted to glutamate, which is then converted to alpha-ketoglutarate, an intermediate product of the TCA cycle [64,65]. Quantitative proteomic analysis revealed that the downregulation of glutamate synthase, glutamate dehydrogenase (GDH), and GS following the treatment of *Fusarium oxysporum* f. sp. *cucumerinum* with canthin-6-one isolated from the tree of heaven (*Ailanthus altissima*) may decrease glutamate and glutamine. The reduction in glutamate and glutamine directly disrupts the biosynthesis of purines and pyrimidines, further affecting the metabolism of nucleic acids [66]. Thus, SPA can significantly regulate the expression of several proteins in *P. neglecta*, thereby disrupting the normal TCA cycle and interfering with energy metabolism. These consequences may further impair the metabolism of mitochondria, nucleic acids, or other substances, leading to the death of the pathogen. The difference is that acetyl-CoA, the starting material for the TCA cycle, is primarily derived from the degradation of monosaccharides and fatty acids. Pyruvate is a product of glycolysis and is converted to acetyl-CoA by the pyruvate dehydrogenase complex (PDC) [60]. After treating *P. neglecta* with SPA, the conversion of acetyl-CoA was promoted by the upregulation of PDC due to the inhibition of the glycolytic pathway to ensure energy metabolism in the pathogen. This may be a response of *P. neglecta* to SPA stress.

Lipids are important biological components of membranes and play an important role in biological processes [67]. The metabolism of lipids affects the integrity of cell membranes, and lipid degradation may disrupt cell membranes. In this study, the proteomic analysis showed that lipase expression was upregulated, while phosphatidylglycerol phospholipase C was downregulated. Fungal lipases not only hydrolyze triglycerides into glycerol and free fatty acids but also participate in biological processes, such as the biosynthesis of membrane lipids [68,69]. The proteomics of *Peronophythora litchii* treated with apple polyphenols showed that lipase and phospholipase D were upregulated in the mycelia and hydrolyzed the structural phospholipids and triglycerides, respectively, leading to the loss of membrane structure and integrity of the pathogen [70]. Phospholipases are thought to play an important role in the invasion of host cells [71,72]. Phospholipase C (PLC) is a key factor that affects fungal development and spore formation during pathogen growth and infection and can be used by pathogenic fungi to disrupt plant cell membranes [73]. Fungal PLCs have now been identified to have important roles in pathogenicity [74]. Benzothiadiazole decreased the activities of PLC, causing the inhibition of *Penicillium* on apples [75]. Therefore, SPA may cause damage to the cell membranes of *P. neglecta* by downregulating the expression of PLC, thus reducing the pathogenicity of the fungus. In our previous study, the scanning electron microscopy (SEM) results also confirmed that the SPA treatment resulted in rough, concave, and even distorted mycelial surfaces. Moreover, the hyphae lost their original morphology and the cellular structure was damaged [41]. Fatty acids are major parts of lipids, and they act as major components of fungal membranes and key metabolites. Thus, they are closely associated with fungal growth and involved in the β-oxidation of energy, cell membrane composition, and stress resistance [76,77,78]. Fatty acid degradation occurs in the characteristic cycle of β-oxidation and produces acetyl-CoA, which is further metabolized to obtain energy and cell biosynthesis precursors. Cinnamaldehyde altered the expression of proteins associated with fatty acid metabolism in *Phytophthora capsici* to inhibit its growth [79]. In this study, SPA altered the expression of proteins associated with fatty acid metabolism, such as acyl-CoA dehydrogenase and acyl-CoA synthetase, in *P. neglecta*. This may have downregulated the degradation of fatty acids, leading to an increase in the contents of fatty acids. After a proteomic analysis of the protein profiles before and after the treatment of *Phytophthora sojae*, knocking out the *PsCPT* gene from the genome of *P. sojae* induced the accumulation of fatty acids. This blocked the transport of fatty acids to the mitochondria and the provision of energy for β-oxidation, and the lack of energy supply reduced the pathogenicity of *P. sojae* [80]. Consequently, the results of this study indicate that SPA regulates fatty acid metabolism in *P. neglecta*, destabilizes cell membranes, and affects the β-oxidation of fatty acids, thereby reducing the energy supply of the pathogen and reducing its pathogenicity.

**Figure 10:**
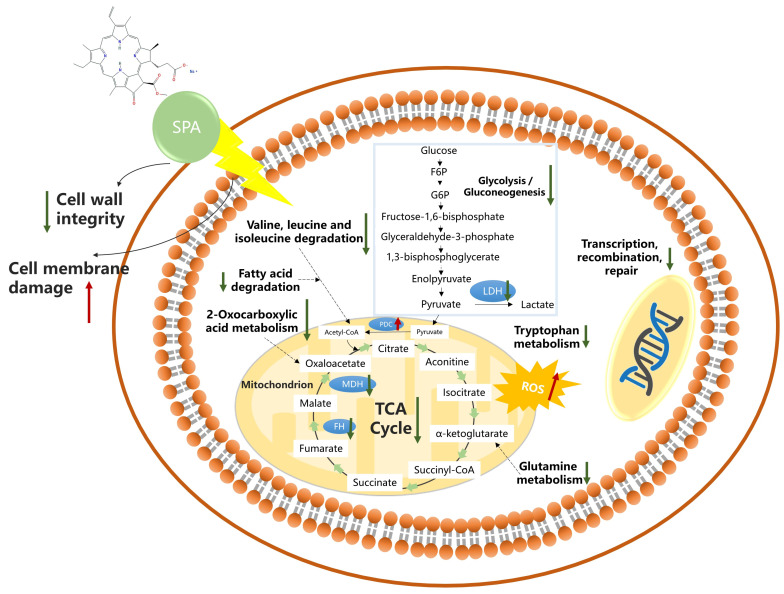
A model that summarizes the antifungal mechanisms of SPA against the *Pestalotiopsis neglecta* mycelia. Green arrows indicate downregulation, while the red arrows show upregulation. SPA, sodium pheophorbide a.

In this study, a hypothetical model of the inhibitory mechanism of SPA against *P. neglecta* was established by performing a high-throughput TMT proteomics analysis of *P. neglecta* treated with SPA in combination with our previous studies (Figure 10). Upon activation by light, SPA produces large amounts of ROS while blocking the expression of genes related to cell integrity, mitochondrial function, and the respiratory chain, and the resulting mitochondrial dysfunction triggers an oxidative stress response. In addition, SPA affects the genes and proteins involved in processing genetic information, including the pathways related to DNA replication, transcription, and repair, through a type I photoreaction. The disruption of these essential functions eventually leads to cell death. This model explains the possible antifungal activity mechanism of SPA against *P. neglecta*.

## 5. Conclusions

The results of this study confirmed that SPA can control black spot needle blight of *P. sylvestris* var. *mongolica* caused by *P. neglecta*. The proteomic analyses further elucidated that SPA may affect the growth and pathogenicity of *P. neglecta* by impairing protein function and related pathways. These findings have practical implications for the future use of SPA to control fungal diseases and provide a new direction for research on novel environmentally friendly fungicides to reduce forest diseases.

## Data Availability

Data are contained within the article.

## Supplementary Materials

The following supporting information can be downloaded at: https://www.mdpi.com/article/10.3390/jof10020102/s1, Table S1: Differentially expressed proteins in *Pestalotiopsis neglecta* after treatment with SPA.

## References

[1] Zheng X., Zhu J.J., Yan Q.L., Song L.N. (2012). Effects of land use changes on the groundwater table and the decline of *Pinus sylvestris* var. *mongolica* plantations in southern Horqin Sandy Land, Northeast China. Agric. Water Manag..

[2] Zhang S.-B., Tian M.-H., Yu H.-L., Hu M.-X., Wang C.-B., Liu W. (2017). Analysis of the Development Status and Characteristics of China’s Trade in Woody Forest Products. Issues For. Econ..

[3] Zhang Y.-C. (2021). Research on the cultivation technology of northern *Pinus sylvestris* var. *mongolica*. Heilongjiang Grain.

[4] Chen C.-Q., Zhang B., Yang L.-N., Gao J. (2011). ldentification and Biological Characteristics of Round Leaf Spot on Blueberry Caused by *Pestalotiopsis photiniae*. J. Northeast. For. Univ..

[5] Evidente A., Zonno M.C., Andolfi A., Troise C., Cimmino A., Vurro M. (2012). Phytotoxic alpha-pyrones produced by *Pestalotiopsis guepinii*, the causal agent of hazelnut twig blight. J. Antibiot..

[6] Fang L., Wang H.R., Feng J.J. (2013). Branch Blight of Loquat Caused by *Pestalotiopsis sydowiana* in China. Plant Dis..

[7] Ismail A.M., Cirvilleri G., Polizzi G. (2013). Characterisation and pathogenicity of *Pestalotiopsis uvicola* and *Pestalotiopsis clavispora* causing grey leaf spot of mango (*Mangifera indica* L.) in Italy. Eur. J. Plant Pathol..

[8] Joshi S.D., Sanjay R., Baby U.I., Mandal A.K.A. (2009). Molecular characterization of *Pestalotiopsis* spp. associated with tea (*Camellia sinensis*) in southern India using RAPD and ISSR markers. Indian J. Biotechnol..

[9] Xu S., Qi Z.-H., Ju H.-C., Feng J.-H., Zhang Y.-Z., Ji L.-H. (1999). Pathogen diagnosis and virogenic study of black spot disease in bagged pear. China Fruits.

[10] Espinoza J.G., Briceno E.X., Keith L.M., Latorre B.A. (2008). Canker and twig dieback of blueberry caused by *Pestalotiopsis* spp. and a *Truncatella* sp. in Chile. Plant Dis..

[11] Qi M., Xie C.-X., Chen Q.-W., Yu Z.-D. (2021). *Pestalotiopsis trachicarpicola*, a novel pathogen causes twig blight of *Pinus bungeana* (Pinaceae: Pinoideae) in China. Antonie Van Leeuwenhoek J. Microbiol..

[12] Chen J., Hao X., Liu X.-F., Ma L. (2020). First Report of *Pestalotiopsis neglecta* Causing Black Spot Needle Blight of *Pinus sylvestris* var. mongolica in China. Plant Dis..

[13] Han Y.-C., Zeng X.-G., Guo C., Zhang Q.-H., Chen F.-Y., Ren L., Chen W.-D., Qin L. (2022). Reproduction response of *Colletotrichum* fungi under the fungicide stress reveals new aspects of chemical control of fungal diseases. Microb. Biotechnol..

[14] Huang C.-D., Hu X.-S., Liao X.-J., Wu J.H. (2007). The advancement of chlorophyll. China Food Addit..

[15] Ding Y., Wu G., Guo C.-K. (2016). Research advance on chlorophyll degradation in plants. Biotechnol. Bull..

[16] Bayona A.M.D.P., Mroz P., Thunshelle C., Hamblin M.R. (2017). Design features for optimization of tetrapyrrole macrocycles as antimicrobial and anticancer photosensitizers. Chem. Biol. Drug Des..

[17] Wang J.-J., Wu X.-R. (2012). Research progress of chlorophyll photosensitizers used in photodynamic therapy. Univ. Chem..

[18] Takamiya K.I., Tsuchiya T., Ohta H. (2000). Degradation pathway(s) of chlorophyll: What has gene cloning revealed?. Trends Plant Sci..

[19] Hortensteiner S. (1999). Chlorophyll breakdown in higher plants and algae. Cell. Mol. Life Sci. CMLS.

[20] Okai Y., Higashi-Okai K. (1997). Potent anti-inflammatory activity of pheophytin a derived from edible green alga, *Enteromorpha prolifera* (Sujiao-nori). Int. J. Immunopharmacol..

[21] Tang L., Xu L., Han Y., Mao H. (2008). Preparation and photosensitive antimicrobial activity of pheophorbide. Chin. J. Antibiot..

[22] Ratnoglik S.L., Aoki C., Sudarmono P., Komoto M., Deng L., Shoji I., Fuchino H., Kawahara N., Hotta H. (2014). Antiviral activity of extracts from *Morinda citrifolia* leaves and chlorophyll catabolites, pheophorbide a and pyropheophorbide a, against hepatitis C virus. Microbiol. Immunol..

[23] Ji J.-Y., Yang J., Zhang B.-W., Wang S.-R., Zhang G.-C., Lin L.-N. (2020). Sodium pheophorbide a controls cherry tomato gray mold (*Botrytis cinerea*) by destroying fungal cell structure and enhancing disease resistance-related enzyme activities in fruit. Pestic. Biochem. Physiol..

[24] Shi L., Liu B., Wei Q., Ge B., Zhang K. (2020). Genome-wide transcriptomic analysis of the response of *Botrytis cinerea* to wuyiencin. PLoS ONE.

[25] Sui Y., Ma Z., Meng X. (2019). Proteomic analysis of the inhibitory effect of oligochitosan on the fungal pathogen, *Botrytis cinerea*. J. Sci. Food Agric..

[26] Li X., Wang Q., Li H., Wang X., Zhang R., Yang X., Jiang Q., Shi Q. (2023). Revealing the Mechanisms for Linalool Antifungal Activity against *Fusarium oxysporum* and Its Efficient Control of Fusarium Wilt in Tomato Plants. Int. J. Mol. Sci..

[27] Yang J., Ji J.-Y., Zhang B.-W., Chen Y.-Z., Wang S.-R., Zhang G.-C., Zhang J. (2020). Transcriptome and cell wall degrading enzyme-related gene analysis of *Pestalotiopsis neglecta* in response to sodium pheophorbide a. Pestic. Biochem. Physiol..

[28] Gotz S., Garcia-Gomez J.M., Terol J., Williams T.D., Nagaraj S.H., Nueda M.J., Robles M., Talon M., Dopazo J., Conesa A. (2008). High-throughput functional annotation and data mining with the Blast2GO suite. Nucleic Acids Res..

[29] Quevillon E., Silventoinen V., Pillai S., Harte N., Mulder N., Apweiler R., Lopez R. (2005). InterProScan: Protein domains identifier. Nucleic Acids Res..

[30] Kanehisa M., Sato Y., Morishima K. (2016). BlastKOALA and GhostKOALA: KEGG Tools for Functional Characterization of Genome and Metagenome Sequences. J. Mol. Biol..

[31] LeBlanc N. (2022). Bacteria in the genus *Streptomyces* are effective biological control agents for management of fungal plant pathogens: A meta-analysis. BioControl.

[32] Deng X., Wang X., Yan X., Ke J., Yang Y., Li Y., Xu D., Zhuo Z., Yan X. (2022). Advances in the Application of Transcriptomics and Metabolomics Analysis to Trees’ Defense Responses to Fungal Diseases. World For. Res..

[33] Aktar M.W., Sengupta D., Chowdhury A. (2009). Impact of pesticides use in agriculture: Their benefits and hazards. Interdiscip. Toxicol..

[34] Sharma A., Shukla A., Attri K., Kumar M., Kumar P., Suttee A., Singh G., Barnwal R.P., Singla N. (2020). Global trends in pesticides: A looming threat and viable alternatives. Ecotoxicol. Environ. Saf..

[35] Yoon M.-Y., Cha B., Kim J.-C. (2013). Recent Trends in Studies on Botanical Fungicides in Agriculture. Plant Pathol. J..

[36] Singh D.J., Singh V.K., Singh D.K. (2017). Photomediated Larvicidal Activity of Pheophorbide a against Cercaria Larvae of *Fasciola gigantica*. Scientifica.

[37] Mittelberger C., Yalcinkaya H., Pichler C., Gasser J., Scherzer G., Erhar T., Schumacher S., Holzner B., Janik K., Robatscher P. (2017). Pathogen-Induced Leaf Chlorosis: Products of Chlorophyll Breakdown Found in Degreened Leaves of Phytoplasma-Infected Apple (*Malus* × *domestica* Borkh.) and Apricot (*Prunus armeniaca* L.) Trees Relate to the Pheophorbide a Oxygenase/Phyllobilin Pathway. J. Agric. Food. Chem..

[38] Hamblin M.R. (2016). Antimicrobial photodynamic inactivation: A bright new technique to kill resistant microbes. Curr. Opin. Microbiol..

[39] Nagai Y., Aizawa S., Iriuchishima T., Goto B., Nagaoka M., Tokuhashi Y., Saito A. (2014). Phototoxic Effect of Na-Pheophorbide A Toward Osteosarcoma Cells in Vitro Using a Laser Diode. Photomed. Laser Surg..

[40] Cai L.-Y., Yang J., Xiong Y.-M., Zhang G.-C., Lin L.-N. (2019). Photosensitive inhibition of sodium pheophorbide a against *Pestalotiopsis neglecta* under different light conditions. Chin. J. Pestic. Sci..

[41] Yang J., Lin L.-N., Zhang X.-B., Wu Y., Bi B., Zhang G.-C., Zou C.-S. (2019). Sodium pheophorbide a has photoactivated fungicidal activity against *Pestalotiopsis neglecta*. Pestic. Biochem. Physiol..

[42] Li Y.-Q., Chen M. (2015). Novel chlorophylls and new directions in photosynthesis research. Funct. Plant Biol..

[43] Melicher P., Dvorak P., Samaj J., Takac T. (2022). Protein-protein interactions in plant antioxidant defense. Front. Plant Sci..

[44] Lehmann S., Serrano M., L’Haridon F., Tjamos S.E., Metraux J.-P. (2015). Reactive oxygen species and plant resistance to fungal pathogens. Phytochemistry.

[45] Petrov V., Hille J., Mueller-Roeber B., Gechev T.S. (2015). ROS-mediated abiotic stress-induced programmed cell death in plants. Front. Plant Sci..

[46] Zhao T.-H., Sun J.-W., Fu Y. (2008). Advances of Research on Metabolism of Plant Reactive Oxygen Species and Exogenous Regulation under Abiotic Stress. Crops.

[47] Al-Issawi M., Rihan H.Z., Al-Shmgani H., Fuller M.P. (2016). Molybdenum application enhances antioxidant enzyme activity and COR15a protein expression under cold stress in wheat. J. Plant Interact..

[48] Haeusler R.E., Ludewig F., Krueger S. (2014). Amino acids—A life between metabolism and signaling. Plant Sci..

[49] Wang Y., Lin W., Yan H., Neng J., Zheng Y., Yang K., Xing F., Sun P. (2021). iTRAQ proteome analysis of the antifungal mechanism of citral on mycelial growth and OTA production in *Aspergillus ochraceus*. J. Sci. Food Agric..

[50] Nishida I., Watanabe D., Tsolmonbaatar A., Kaino T., Ohtsu I., Takagi H. (2016). Vacuolar amino acid transporters upregulated by exogenous proline and involved in cellular localization of proline in *Saccharomyces cerevisiae*. J. Gen. Appl. Microbiol..

[51] McCarthy M.W., Walsh T.J. (2018). Amino Acid Metabolism and Transport Mechanisms as Potential Antifungal Targets. Int. J. Mol. Sci..

[52] Luo F., Zhou H., Zhou X., Xie X., Li Y., Hu F., Huang B. (2020). The Intermediates in Branched-Chain Amino Acid Biosynthesis Are Indispensable for Conidial Germination of the Insect-Pathogenic Fungus *Metarhizium robertsii*. Appl. Environ. Microbiol..

[53] Rodriguez-Frometa R.A., Gutierrez A., Torres-Martinez S., Garre V. (2013). Malic enzyme activity is not the only bottleneck for lipid accumulation in the oleaginous fungus *Mucor circinelloides*. Appl. Microbiol. Biotechnol..

[54] Downes D.J., Dayis M.A., Kreutzberger S.D., Taig B.L., Todd R.B. (2013). Regulation of the NADP-glutamate dehydrogenase gene gdhA in *Aspergillus nidulans* by the Zn(II)2Cys6 transcription factor LeuB. Microbiology.

[55] Wang P., Chang P.-K., Kong Q., Shan S., Wei Q. (2019). Comparison of aflatoxin production of *Aspergillus flavus* at different temperatures and media: Proteome analysis based on TMT. Int. J. Food Microbiol..

[56] Guo M., Liu J., Xu Z., Wang J., Li T., Lei H., Duan X., Sun Y., Zhang X., Huang R. (2020). 2-Methoxy-1,4-naphthoquinone Induces Metabolic Shifts in Penicillium Digitatum Revealed by High-Dimensional Biological Data. J. Agric. Food. Chem..

[57] You J., Zhang Y., Liu A., Li D., Wang X., Dossa K., Zhou R., Yu J., Zhang Y., Wang L. (2019). Transcriptomic and metabolomic profiling of drought-tolerant and susceptible sesame genotypes in response to drought stress. BMC Plant Biol..

[58] Shaughnessy D.T., McAllister K., Worth L., Haugen A.C., Meyer J.N., Domann F.E., Van Houten B., Mostoslavsky R., Bultman S.J., Baccarelli A.A. (2014). Mitochondria, Energetics, Epigenetics, and Cellular Responses to Stress. Environ. Health Perspect..

[59] Cui K., He L., Zhao Y., Mu W., Lin J., Liu F. (2021). Comparative Analysis of *Botrytis cinerea* in Response to the Microbial Secondary Metabolite Benzothiazole Using iTRAQ-Based Quandiative Proteomics. Phytopathology.

[60] Hu Z., Lu C., Zhang Y., Tong W., Du L., Liu F. (2022). Proteomic analysis of Aspergillus flavus reveals the antifungal action of Perilla frutescens essential oil by interfering with energy metabolism and defense function. LWT Food Sci. Technol..

[61] Wang N., Shao X., Wei Y., Jiang S., Xu F., Wang H. (2020). Quantitative proteomics reveals that tea tree oil effects *Botrytis cinerea* mitochondria function. Pestic. Biochem. Physiol..

[62] Yang S., Liu L., Li D., Xia H., Su X., Peng L., Pan S. (2016). Use of active extracts of poplar buds against *Penicillium italicum* and possible modes of action. Food Chem..

[63] Xu J., Shao X., Wei Y., Xu F., Wang H. (2017). iTRAQ Proteomic Analysis Reveals That Metabolic Pathways Involving Energy Metabolism Are Affected by Tea Tree Oil in *Botrytis cinerea*. Front. Microbiol..

[64] Canas R.A., Quillere I., Lea P.J., Hirel B. (2010). Analysis of amino acid metabolism in the ear of maize mutants deficient in two cytosolic glutamine synthetase isoenzymes highlights the importance of asparagine for nitrogen translocation within sink organs. Plant Biotechnol. J..

[65] Margelis S., D’Souza C., Small A.J., Hynes M.J., Adams T.H., Davis M.A. (2001). Role of glutamine synthetase in nitrogen metabolite repression in Aspergillus nidulans. J. Bacteriol..

[66] Li Y., Zhao M., Zhang Z. (2021). Quantitative proteomics reveals the antifungal effect of canthin-6-one isolated from *Ailanthus altissima* against *Fusarium oxysporum* f. sp. *cucumerinum* in vitro. PLoS ONE.

[67] Stahl A., Gimeno R.E., Tartaglia L.A., Lodish H.F. (2001). Fatty acid transport proteins: A current view of a growing family. Trends Endocrinol. Metab..

[68] Kim S., Lee J., Park J., Choi S., Bui D.-C., Kim J.-E., Shin J., Kim H., Choi G.J., Lee Y.-W. (2023). Genetic and Transcriptional Regulatory Mechanisms of Lipase Activity in the Plant Pathogenic Fungus *Fusarium graminearum*. Microbiol. Spectrum.

[69] Zanghellini J., Natter K., Jungreuthmayer C., Thalhammer A., Kurat C.F., Gogg-Fassolter G., Kohlwein S.D., von Gruenberg H.-H. (2008). Quantitative modeling of triacylglycerol homeostasis in yeast–metabolic requirement for lipolysis to promote membrane lipid synthesis and cellular growth. FEBS J..

[70] Xiang Y., Su Z., Li T., Xu D., Hu M., Sun J., Jiang Y., Zhang Z. (2022). Inhibitory activity and action mode of apple polyphenols against *Peronophythora litchii* that causes litchi downy blight. Postharvest Biol. Technol..

[71] Mayser P., Laabs S., Heuer K.U., Grunder K. (1996). Detection of extracellular phospholipase activity in *Candida albicans* and *Rhodotorula rubra*. Mycopathologia.

[72] Ibrahim A.S., Mirbod F., Filler S.G., Banno Y., Cole G.T., Kitajima Y., Edwards J.E., Nozawa Y., Ghannoum M.A. (1995). Evidence implicating phospholipase as a virulence factor of *Candida albicans*. Infect. Immun..

[73] Fankhauser H., Schweingruber A.M., Edenharter E., Schweingruber M.E. (1995). Growth of a mutant defective in a putative phosphoinositide-specific phospholipase C of *Schizosaccharomyces pombe* is restored by low concentrations of phosphate and inositol. Curr. Genet..

[74] Fang Y., Jiang J., Ding H., Li X., Xie X. (2023). Phospholipase C: Diverse functions in plant biotic stress resistance and fungal pathogenicity. Mol. Plant Pathol.

[75] Gong D., Bi Y., Zhang X., Han Z., Zong Y., Li Y., Sionov E., Prusky D. (2021). Benzothiadiazole treatment inhibits membrane lipid metabolism and straight-chain volatile compound release in *Penicillium expansum*-inoculated apple fruit. Postharvest Biol. Technol..

[76] Pang Z., Chen L., Mu W., Liu L., Liu X. (2016). Insights into the adaptive response of the plant-pathogenic oomycete *Phytophthora capsici* to the fungicide flumorph. Sci. Rep..

[77] Pang Z., Chen L., Miao J., Wang Z., Bulone V., Liu X. (2015). Proteomic profile of the plant-pathogenic oomycete *Phytophthora capsici* in response to the fungicide pyrimorph. Proteomics.

[78] Calvo A.M., Gardner H.W., Keller N.P. (2001). Genetic connection between fatty acid metabolism and sporulation in *Aspergillus nidulans*. J. Biol. Chem..

[79] Wang Y.-N., Wang M.-K., Li M., Zhao T., Zhou L. (2021). Cinnamaldehyde inhibits the growth of *Phytophthora capsici* through disturbing metabolic homoeostasis. PeerJ.

[80] Liu D., Pan Y., Li K., Li D., Li P., Gao Z. (2020). Proteomics Reveals the Mechanism Underlying the Inhibition of *Phytophthora sojae* by Propyl Gallate. J. Agric. Food. Chem..

