# Supplementary material for: TMT-Based Proteomic Analysis Reveals the Molecular Mechanisms of Sodium Pheophorbide A against Black Spot Needle Blight Caused by Pestalotiopsis neglecta in Pinus sylvestris var. mongolica
Source: J Fungi (Basel). 2024 Jan 26;10(2):102. doi: 10.3390/jof10020102 (PMC10889695; doi:10.3390/jof10020102)
Supplement: Supplementary file 1 [file jof-10-00102-s001.zip › jof-2821549-supplementary.pdf]

Journal of Fungi

MDPI

# Supplemetray Materials

**Table S1.** Differentially expressed proteins in *P. neglecta* after treatment with SPA.

| Accession | Sequence Description | Score | Coverage [%] | Fold change | *P*-value |
|---|---|---|---|---|---|
| Gene.10060::TRINITY_DN16389_c0_g1::g.10060::m.10060 | 40S ribosomal protein S20 | 157.918 | 47 | 0.83094 | 0.01302 |
| Gene.101::TRINITY_DN10172_c0_g1::g.101::m.101 | 40S ribosomal protein S16 | 295.819 | 7 | 0.79533 | 0.01735 |
| Gene.10267::TRINITY_DN16452_c0_g2::g.10267::m.10267 | Benzoate 4-monooxygenase | 623.239 | 4 | 1.50983 | 0.02603 |
| Gene.10322::TRINITY_DN16467_c0_g1::g.10322::m.10322 | Sexual differentiation process protein isp4 | 584.719 | 5 | 1.73394 | 0.03287 |
| Gene.10367::TRINITY_DN16488_c0_g3::g.10367::m.10367 | Cyclic pyranopterin monophosphate synthase, mitochondrial | 157.532 | 4 | 1.22095 | 0.03621 |
| Gene.10394::TRINITY_DN16500_c0_g1::g.10394::m.10394 | Bifunctional cytochrome P450 / NADPH-P450 reductase | 1315.06 | 20 | 1.45399 | 0.02816 |
| Gene.10420::TRINITY_DN16509_c0_g1::g.10420::m.10420 | Neutrophil cytosol factor 2 | 147.132 | 6 | 1.4777 | 0.03805 |
| Gene.10479::TRINITY_DN16533_c0_g2::g.10479::m.10479 | Deoxyuridine 5'-triphosphate nucleotidohydrolase | 228.024 | 37 | 0.69252 | 0.00487 |
| Gene.10500::TRINITY_DN16540_c0_g2::g.10500::m.10500 | Vacuolar morphogenesis protein 6 | 171.4 | 8 | 1.23048 | 0.00953 |
| Gene.10529::TRINITY_DN16547_c0_g1::g.10529::m.10529 | Actin-related protein 2/3 complex subunit 3 | 138.658 | 4 | 0.77755 | 0.00885 |
| Gene.1064::TRINITY_DN11092_c1_g5::g.1064::m.1064 | Flavohemoprotein A | 259.225 | 64 | 0.62014 | 0.007 |
| Gene.10646::TRINITY_DN16581_c0_g1::g.10646::m.10646 | GPI-anchored CFEM domain protein ARB_01545 | 58.5362 | 6 | 1.99401 | 0.03057 |
| Gene.10655::TRINITY_DN16584_c0_g1::g.10655::m.10655 | Clustered mitochondria protein homolog | 1596.64 | 50 | 0.77096 | 0.0245 |
| Gene.10989::TRINITY_DN16697_c0_g1::g.10989::m.10989 | Cyclic AMP-dependent transcription factor ATF-2 | 97.8265 | 8 | 0.76626 | 0.01514 |
| Gene.11119::TRINITY_DN16746_c0_g2::g.11119::m.11119 | ATP synthase subunit delta, mitochondrial | 272.707 | 28 | 0.657 | 0.00967 |
| Gene.11200::TRINITY_DN16770_c0_g3::g.11200::m.11200 | Activator of stress genes 1 | 280.026 | 3 | 1.29308 | 0.01491 |
| Gene.11294::TRINITY_DN16802_c0_g1::g.11294::m.11294 | 40S ribosomal protein S2 | 590.882 | 8 | 1.25432 | 0.02736 |
| Gene.11570::TRINITY_DN16893_c0_g1::g.11570::m.11570 | BolA-like protein 1 | 91.2781 | 20 | 0.74043 | 0.03728 |
| Gene.1158::TRINITY_DN11156_c0_g1::g.1158::m.1158 | Pre-rRNA-processing protein, PNO1 | 371.703 | 14 | 0.75879 | 0.03031 |
| Gene.11689::TRINITY_DN16928_c0_g3::g.11689::m.11689 | Glucan endo-1,3-alpha-glucosidase, agn1 | 206.068 | 17 | 1.25225 | 0.03723 |
| Gene.11768::TRINITY_DN16953_c0_g2::g.11768::m.11768 | SWI/SNF chromatin-remodeling complex subunit, sol1 | 176.022 | 7 | 0.77253 | 0.00105 |
| Gene.11850::TRINITY_DN16978_c0_g1::g.11850::m.11850 | Vegetative incompatibility protein, HET-E-1 | 60.8474 | 58 | 0.71674 | 0.01031 |
| Gene.11946::TRINITY_DN16993_c0_g1::g.11946::m.11946 | N-alpha-acetyltransferase 30 | 161.384 | 17 | 1.97471 | 0.04182 |
| Gene.12075::TRINITY_DN17038_c0_g2::g.12075::m.12075 | 54S ribosomal protein L12, mitochondrial | 128.257 | 38 | 0.56878 | 0.01654 |
| Gene.12096::TRINITY_DN17043_c0_g1::g.12096::m.12096 | Protein BCP1 | 353.214 | 38 | 0.8229 | 0.03779 |
| Gene.12114::TRINITY_DN17046_c0_g4::g.12114::m.12114 | Guanine deaminase | 80.1073 | 9 | 1.6404 | 0.00122 |
| Gene.12223::TRINITY_DN17085_c0_g1::g.12223::m.12223 | Regulator of V-ATPase in vacuolar membrane protein 2 | 44.2838 | 8 | 1.2805 | 0.00042 |
| Gene.12281::TRINITY_DN17100_c1_g2::g.12281::m.12281 | Probable DNA primase large subunit | 749.199 | 5 | 0.79319 | 0.03209 |
| Gene.12414::TRINITY_DN17133_c0_g3::g.12414::m.12414 | mRNA export factor rsm1 | 72.0182 | 2 | 0.71478 | 0.00086 |
| Gene.12419::TRINITY_DN17137_c0_g2::g.12419::m.12419 | Calcium-binding protein NCS-1 | 321.627 | 40 | 0.75431 | 0.00669 |
| Gene.12493::TRINITY_DN1715_c0_g1::g.12493::m.12493 | Probable proteasome subunit beta type-2 | 348.977 | 17 | 0.68161 | 0.01104 |
| Gene.12606::TRINITY_DN17194_c1_g4::g.12606::m.12606 | Mitochondrial fission 1 protein | 252.292 | 27 | 0.638 | 0.0188 |
| Gene.12607::TRINITY_DN17195_c0_g2::g.12607::m.12607 | Probable D-xylulose reductase A | 115.931 | 24 | 1.82134 | 0.02358 |
| Gene.12867::TRINITY_DN17252_c0_g2::g.12867::m.12867 | Bleomycin hydrolase | 138.658 | 11 | 0.70891 | 0.00626 |
| Gene.12872::TRINITY_DN17253_c0_g1::g.12872::m.12872 | Sphingolipid long chain base-responsive protein PIL1 | 190.274 | 60 | 1.50585 | 0.03836 |
| Gene.12894::TRINITY_DN17257_c0_g1::g.12894::m.12894 | UBX domain-containing protein 1 | 107.071 | 35 | 0.79533 | 0.02442 |
| Gene.12944::TRINITY_DN17272_c1_g1::g.12944::m.12944 | Single-stranded TG1-3 DNA-binding protein | 124.79 | 63 | 0.79211 | 0.03038 |

| | | | | | |
|---|---|---|---|---|---|
| Gene.13076::TRINITY_DN17309_c0_g1::g.13076::m.13076 | RING finger protein ETP1 homolog | 313.538 | 2 | 1.23714 | 0.01577 |
| Gene.13103::TRINITY_DN17314_c0_g1::g.13103::m.13103 | Uncharacterized oxidoreductase | 208.764 | 69 | 0.70746 | 0.00308 |
| Gene.13204::TRINITY_DN17341_c0_g1::g.13204::m.13204 | Probable eukaryotic translation initiation factor 2 subunit beta | 227.254 | 50 | 0.8276 | 0.01538 |
| Gene.13208::TRINITY_DN17342_c0_g2::g.13208::m.13208 | Probable fumarate hydratase, mitochondrial | 669.848 | 58 | 0.81464 | 0.02774 |
| Gene.13435::TRINITY_DN17399_c0_g1::g.13435::m.13435 | Myosin regulatory light chain cdc4 | 181.03 | 83 | 0.76991 | 0.02569 |
| Gene.13462::TRINITY_DN17404_c0_g5::g.13462::m.13462 | Putative glycine cleavage system H protein, mitochondrial | 116.316 | 39 | 0.62839 | 0.00105 |
| Gene.13489::TRINITY_DN17410_c0_g2::g.13489::m.13489 | Hit family protein 1 | 72.4034 | 16 | 0.77148 | 0.04045 |
| Gene.13507::TRINITY_DN17416_c0_g1::g.13507::m.13507 | NAD(P)H-dependent D-xylose reductase, xyl1 | 255.758 | 4 | 1.2779 | 0.02825 |
| Gene.13533::TRINITY_DN17418_c1_g8::g.13533::m.13533 | Ammonium transporter 1 | 497.278 | 3 | 2.44687 | 0.00833 |
| Gene.1380::TRINITY_DN11320_c1_g1::g.1380::m.1380 | Methyltransferase, ustM | 128.257 | 28 | 1.28311 | 0.00646 |
| Gene.13831::TRINITY_DN17504_c0_g1::g.13831::m.13831 | Xanthocillin biosynthesis cluster protein D | 94.7449 | 12 | 1.22845 | 0.0067 |
| Gene.13904::TRINITY_DN17526_c0_g1::g.13904::m.13904 | NADH-dependent flavin oxidoreductase, nadA | 405.601 | 29 | 1.25141 | 0.00979 |
| Gene.14155::TRINITY_DN17584_c0_g3::g.14155::m.14155 | NA polymerase-associated protein C651.09c | 126.716 | 7 | 1.24757 | 0.00202 |
| Gene.14393::TRINITY_DN17631_c0_g3::g.14393::m.14393 | Protein IVY1 | 102.449 | 5 | 1.23547 | 0.04438 |
| Gene.14578::TRINITY_DN17679_c0_g3::g.14578::m.14578 | Secretory component protein SHR3 | 60.4622 | 29 | 1.32919 | 0.03613 |
| Gene.14582::TRINITY_DN1767_c0_g2::g.14582::m.14582 | Mitochondrial import receptor subunit tom22 | 149.058 | 28 | 0.63177 | 0.01677 |
| Gene.14686::TRINITY_DN17702_c0_g8::g.14686::m.14686 | Mitochondrial protein cyt-4 | 628.632 | 9 | 0.75182 | 0.01388 |
| Gene.14752::TRINITY_DN17717_c0_g1::g.14752::m.14752 | Phosphatidylinositol 4,5-bisphosphate-binding protein | 300.056 | 16 | 1.24551 | 0.00186 |
| Gene.14900::TRINITY_DN17742_c0_g4::g.14900::m.14900 | Ferrochelatase, mitochondrial | 426.017 | 41 | 0.82815 | 0.0232 |
| Gene.15070::TRINITY_DN17778_c0_g1::g.15070::m.15070 | Protein transport protein SEC61 subunit alpha | 935.636 | 14 | 1.41895 | 0.00325 |
| Gene.15106::TRINITY_DN17785_c1_g5::g.15106::m.15106 | Ornithine aminotransferase | 642.884 | 52 | 0.7613 | 0.02381 |
| Gene.15162::TRINITY_DN17799_c0_g1::g.15162::m.15162 | Phosphatidylglycerol/phosphatidylinositol transfer protein | 189.504 | 42 | 1.3261 | 0.04914 |
| Gene.15167::TRINITY_DN17800_c0_g1::g.15167::m.15167 | 40S ribosomal protein S21 | 154.451 | 45 | 1.31303 | 0.00863 |
| Gene.15236::TRINITY_DN17818_c0_g1::g.15236::m.15236 | Methionine aminopeptidase 1 | 428.328 | 38 | 0.78991 | 0.00232 |
| Gene.15286::TRINITY_DN17832_c0_g2::g.15286::m.15286 | 3,4-dihydroxy-2-butanone 4-phosphate synthase | 265.388 | 56 | 0.70455 | 0.01971 |
| Gene.15351::TRINITY_DN17839_c0_g1::g.15351::m.15351 | Rho GDP-dissociation inhibitor | 177.563 | 64 | 0.79587 | 0.0314 |
| Gene.15486::TRINITY_DN17870_c0_g1::g.15486::m.15486 | Succinate--CoA ligase [ADP-forming] subunit alpha, mitochondrial | 326.25 | 37 | 0.81683 | 0.00463 |
| Gene.15571::TRINITY_DN17883_c0_g1::g.15571::m.15571 | GTPase-activating protein, GYP7 | 631.713 | 4 | 1.25781 | 0.00185 |
| Gene.15710::TRINITY_DN178_c0_g1::g.15710::m.15710 | ATP synthase assembly factor FMC1, mitochondrial | 51.9878 | 15 | 1.37208 | 0.00185 |
| Gene.15800::TRINITY_DN17919_c0_g3::g.15800::m.15800 | Microsomal glutathione S-transferase 3 | 77.411 | 15 | 1.95081 | 0.00526 |
| Gene.15833::TRINITY_DN17927_c1_g4::g.15833::m.15833 | Glyoxalase 3 | 78.5666 | 25 | 1.58398 | 0.04093 |
| Gene.15852::TRINITY_DN17933_c0_g3::g.15852::m.15852 | Cytochrome c oxidase subunit 6, mitochondrial | 57.3806 | 10 | 0.45278 | 0.00422 |
| Gene.1586::TRINITY_DN11491_c0_g1::g.1586::m.1586 | DNA-directed RNA polymerase II subunit RPB9 | 91.2781 | 3 | 0.75446 | 0.02215 |
| Gene.15860::TRINITY_DN17934_c2_g15::g.15860::m.15860 | Putative 26S proteasome complex subunit sem-1 | 72.4034 | 32 | 0.5064 | 0.02429 |
| Gene.15986::TRINITY_DN17950_c0_g2::g.15986::m.15986 | Tyrosine--tRNA ligase, mitochondrial | 629.402 | 25 | 0.72612 | 0.00879 |
| Gene.16301::TRINITY_DN18014_c1_g3::g.16301::m.16301 | Uncharacterized transcriptional regulatory protein C15D4.02 | 85.1149 | 2 | 1.31036 | 0.00757 |
| Gene.16335::TRINITY_DN18020_c0_g2::g.16335::m.16335 | 40S ribosomal protein S15 | 232.646 | 23 | 0.7194 | 0.00595 |
| Gene.16569::TRINITY_DN18053_c0_g5::g.16569::m.16569 | 2,3-dihydroxybenzoate decarboxylase | 201.06 | 3 | 1.2531 | 0.01843 |
| Gene.16616::TRINITY_DN18059_c0_g2::g.16616::m.16616 | NADH dehydrogenase [ubiquinone] 1 alpha subcomplex assembly factor 3 | 62.003 | 18 | 0.82205 | 0.03211 |
| Gene.16630::TRINITY_DN18061_c0_g1::g.16630::m.16630 | 40S ribosomal protein S28 | 129.028 | 30 | 0.81105 | 0.01723 |
| Gene.16658::TRINITY_DN18066_c1_g12::g.16658::m.16658 | Vacuolar protein sorting-associated protein 41 homolog | 199.904 | 4 | 1.26112 | 0.01993 |
| Gene.1696::TRINITY_DN11594_c0_g1::g.1696::m.1696 | NADH-ubiquinone oxidoreductase 23 kDa subunit, mitochondrial | 359.762 | 41 | 1.25602 | 0.00657 |

| | | | | | |
|---|---|---|---|---|---|
| Gene.17002::TRINITY_DN18107_c1_g1::g.17002::m.17002 | Nuclear distribution protein, PAC1 | 627.476 | 17 | 1.29973 | 0.00398 |
| Gene.17208::TRINITY_DN18131_c1_g9::g.17208::m.17208 | Arginine metabolism regulation protein II | 57.7658 | 2 | 1.66002 | 0.00039 |
| Gene.17295::TRINITY_DN18137_c0_g17::g.17295::m.17295 | Uncharacterized oxidoreductase C26F1.07 | 224.172 | 11 | 1.21239 | 0.01273 |
| Gene.17340::TRINITY_DN18141_c1_g13::g.17340::m.17340 | Phosphatidylserine decarboxylase proenzyme 1, mitochondrial | 384.8 | 16 | 0.78336 | 0.02447 |
| Gene.17343::TRINITY_DN18141_c1_g16::g.17343::m.17343 | Molybdopterin synthase catalytic subunit | 263.077 | 6 | 0.76115 | 0.00371 |
| Gene.17355::TRINITY_DN18141_c1_g7::g.17355::m.17355 | 60 kDa lysophospholipase | 317.775 | 25 | 0.78253 | 0.04835 |
| Gene.17472::TRINITY_DN18150_c0_g7::g.17472::m.17472 | Reticulocyte-binding protein 2 homolog a | 59.6918 | 2 | 0.79647 | 0.03988 |
| Gene.17654::TRINITY_DN18184_c1_g12::g.17654::m.17654 | Transcriptional regulator RPN4 | 93.9745 | 6 | 0.689 | 0.04913 |
| Gene.17713::TRINITY_DN18185_c0_g1::g.17713::m.17713 | UBP9-binding protein, bun107 | 191.045 | 6 | 1.21157 | 0.01472 |
| Gene.17795::TRINITY_DN18190_c1_g1::g.17795::m.17795 | Uncharacterized protein YJL213W | 45.4394 | 16 | 1.34283 | 0.04048 |
| Gene.17797::TRINITY_DN18190_c1_g10::g.17797::m.17797 | Uncharacterized protein PYUK71.03c | 668.692 | 25 | 1.24757 | 0.01714 |
| Gene.17835::TRINITY_DN18191_c1_g1::g.17835::m.17835 | Nucleoporin POM33 | 350.903 | 27 | 1.25094 | 0.03085 |
| Gene.17838::TRINITY_DN18191_c1_g4::g.17838::m.17838 | Temperature-induced lipocalin-1 | 59.3066 | 24 | 9.10101 | 0.00538 |
| Gene.17875::TRINITY_DN18199_c0_g10::g.17875::m.17875 | Copper amine oxidase 1 | 861.677 | 29 | 1.39004 | 0.00066 |
| Gene.17879::TRINITY_DN18199_c0_g2::g.17879::m.17879 | Splicing factor 3B subunit 4 | 267.314 | 5 | 0.77357 | 0.01656 |
| Gene.17904::TRINITY_DN18200_c1_g1::g.17904::m.17904 | 60S ribosomal protein L23-B | 241.506 | 17 | 1.68261 | 0.00808 |
| Gene.17937::TRINITY_DN18205_c3_g16::g.17937::m.17937 | Pyridoxal phosphate homeostasis protein | 201.06 | 27 | 0.74469 | 0.00248 |
| Gene.18::TRINITY_DN10041_c0_g1::g.18::m.18 | Ribosome-releasing factor 2, mitochondrial | 597.045 | 5 | 1.23003 | 0.04671 |
| Gene.18091::TRINITY_DN18224_c2_g4::g.18091::m.18091 | Catalase | 613.609 | 21 | 1.67261 | 0.0215 |
| Gene.18227::TRINITY_DN18240_c1_g1::g.18227::m.18227 | NEDD8-specific protease 2 | 105.916 | 5 | 1.21774 | 0.01676 |
| Gene.18345::TRINITY_DN18257_c0_g2::g.18345::m.18345 | 40S ribosomal protein S9 | 163.31 | 65 | 1.3015 | 0.01379 |
| Gene.18346::TRINITY_DN18257_c0_g3::g.18346::m.18346 | 40S ribosomal protein S9-A | 312.768 | 24 | 1.75023 | 0.00137 |
| Gene.18356::TRINITY_DN18258_c0_g2::g.18356::m.18356 | Pinoresinol reductase 2 | 55.0694 | 15 | 1.92826 | 3.8E-05 |
| Gene.18439::TRINITY_DN18267_c2_g7::g.18439::m.18439 | Uncharacterized protein YJL213W | 132.109 | 10 | 1.22103 | 0.03057 |
| Gene.18454::TRINITY_DN18269_c1_g1::g.18454::m.18454 | Uncharacterized membrane protein YJR124C | 263.462 | 14 | 1.32378 | 0.04306 |
| Gene.18573::TRINITY_DN18277_c0_g4::g.18573::m.18573 | Uncharacterized protein C354.08c | 427.557 | 2 | 1.51467 | 0.0031 |
| Gene.1873::TRINITY_DN11773_c0_g1::g.1873::m.1873 | C-1-tetrahydrofolate synthase, cytoplasmic | 1187.17 | 48 | 0.78761 | 0.02697 |
| Gene.18766::TRINITY_DN18291_c0_g2::g.18766::m.18766 | Putative fungistatic metabolite | 58.151 | 14 | 1.70758 | 0.01876 |
| Gene.18956::TRINITY_DN18310_c3_g5::g.18956::m.18956 | Probable dihydrolipoyllysine-residue succinyltransferase component of 2-oxoglutarate dehydrogenase complex, mitochondrial | 481.1 | 47 | 0.79533 | 0.02147 |
| Gene.19092::TRINITY_DN18321_c2_g6::g.19092::m.19092 | Histidine biosynthesis trifunctional protein | 1296.18 | 44 | 0.75901 | 0.00774 |
| Gene.19190::TRINITY_DN18327_c3_g6::g.19190::m.19190 | Mannose-1-phosphate guanyltransferase | 641.728 | 50 | 0.82871 | 0.00242 |
| Gene.19251::TRINITY_DN1832_c0_g1::g.19251::m.19251 | Dicarboxylic amino acid permease | 382.489 | 5 | 1.79367 | 0.02976 |
| Gene.19281::TRINITY_DN18331_c0_g8::g.19281::m.19281 | Probable secreted lipase ARB_02369 | 179.104 | 9 | 1.77778 | 0.01863 |
| Gene.194::TRINITY_DN10320_c0_g1::g.194::m.194 | Peroxisomal multifunctional enzyme type 2 | 100.138 | 7 | 2.52113 | 0.04521 |
| Gene.19591::TRINITY_DN18354_c0_g19::g.19591::m.19591 | Putative glutathione-dependent formaldehyde-activating enzyme | 43.5134 | 7 | 1.47893 | 0.00523 |
| Gene.19635::TRINITY_DN18357_c0_g1::g.19635::m.19635 | Pyruvate decarboxylase | 1047.73 | 6 | 1.56849 | 0.02976 |
| Gene.19663::TRINITY_DN18362_c0_g2::g.19663::m.19663 | Heat shock protein SSC1, mitochondrial | 106.301 | 28 | 2.125 | 0.00674 |
| Gene.1972::TRINITY_DN11828_c0_g2::g.1972::m.1972 | UPF0676 protein C1494.01 | 60.077 | 13 | 1.20832 | 0.03941 |
| Gene.19755::TRINITY_DN18365_c0_g2::g.19755::m.19755 | 60S ribosomal protein L10a | 361.303 | 28 | 0.81159 | 0.04803 |
| Gene.19848::TRINITY_DN18370_c2_g3::g.19848::m.19848 | Protein-lysine N-methyltransferase EFM4 | 157.532 | 8 | 1.537 | 0.0081 |
| Gene.19894::TRINITY_DN18373_c0_g3::g.19894::m.19894 | Heat shock protein 60 | 959.903 | 61 | 0.7369 | 0.03357 |

| | | | | | |
|---|---|---|---|---|---|
| Gene.20005::TRINITY_DN18383_c0_g4::g.20005::m.20005 | Poly [ADP-ribose] polymerase 2 | 326.25 | 12 | 0.74571 | 0.02553 |
| Gene.20174::TRINITY_DN18390_c1_g15::g.20174::m.20174 | Chitobiosyldiphosphodolichol beta-mannosyltransferase | 327.405 | 16 | 1.26112 | 0.00172 |
| Gene.20181::TRINITY_DN18390_c1_g4::g.20181::m.20181 | Uncharacterized J domain-containing protein C3E7.11c | 211.846 | 41 | 0.77515 | 0.00934 |
| Gene.202::TRINITY_DN1032_c0_g1::g.202::m.202 | tRNA-aminoacylation cofactor, ARC1 | 182.185 | 50 | 0.73661 | 0.03453 |
| Gene.20269::TRINITY_DN18396_c0_g2::g.20269::m.20269 | 60S ribosomal protein L12 | 288.886 | 52 | 0.82039 | 0.00721 |
| Gene.20316::TRINITY_DN18400_c0_g5::g.20316::m.20316 | Membrane primary amine oxidase | 297.36 | 36 | 1.80374 | 0.00119 |
| Gene.20425::TRINITY_DN18403_c0_g6::g.20425::m.20425 | Peptide-N4-(N-acetyl-beta-glucosaminyl)asparagine amidase A | 198.364 | 2 | 2.00702 | 0.00811 |
| Gene.20433::TRINITY_DN18404_c0_g2::g.20433::m.20433 | Uncharacterized isomerase YHI9 | 74.7146 | 19 | 0.58746 | 0.00764 |
| Gene.2044::TRINITY_DN11859_c0_g1::g.2044::m.2044 | Probable helicase mot1 | 1607.42 | 12 | 1.22763 | 0.00842 |
| Gene.20649::TRINITY_DN18413_c2_g1::g.20649::m.20649 | Uncharacterized mitochondrial carrier C8C9.12c | 308.531 | 12 | 0.79718 | 0.00371 |
| Gene.20704::TRINITY_DN18416_c0_g2::g.20704::m.20704 | Asparagine synthetase [glutamine-hydrolyzing] 1 | 286.189 | 17 | 0.82365 | 0.01301 |
| Gene.20737::TRINITY_DN18421_c0_g8::g.20737::m.20737 | Uncharacterized protein C13G1.09 | 315.079 | 11 | 0.83008 | 0.02942 |
| Gene.20840::TRINITY_DN18428_c0_g2::g.20840::m.20840 | Transcription factor himD | 74.7146 | 4 | 1.22469 | 0.02631 |
| Gene.20899::TRINITY_DN18431_c0_g3::g.20899::m.20899 | Malate dehydrogenase, mitochondrial | 312.768 | 69 | 0.66159 | 0.02604 |
| Gene.20948::TRINITY_DN18435_c2_g1::g.20948::m.20948 | Uncharacterized RING finger protein P32A8.03c | 120.553 | 5 | 0.80693 | 0.03544 |
| Gene.21021::TRINITY_DN18437_c0_g1::g.21021::m.21021 | Ergothioneine biosynthesis protein 1 | 652.514 | 12 | 0.72414 | 0.00434 |
| Gene.21089::TRINITY_DN18441_c1_g22::g.21089::m.21089 | Aromatic amino acid aminotransferase C1773.13 | 182.185 | 2 | 1.27964 | 0.0013 |
| Gene.21107::TRINITY_DN18441_c1_g7::g.21107::m.21107 | Uncharacterized membrane protein YMR155W | 174.866 | 2 | 1.45741 | 0.04476 |
| Gene.21112::TRINITY_DN18441_c1_g9::g.21112::m.21112 | Beta-1,3-glucan-binding protein | 219.55 | 16 | 1.75482 | 0.01483 |
| Gene.21117::TRINITY_DN18443_c0_g1::g.21117::m.21117 | Protein sfk1 | 73.559 | 4 | 1.30985 | 0.00268 |
| Gene.21160::TRINITY_DN18444_c0_g27::g.21160::m.21160 | Secondary metabolism regulator LAE1 | 277.715 | 5 | 1.731 | 0.02191 |
| Gene.21251::TRINITY_DN18452_c1_g14::g.21251::m.21251 | Universal stress protein A family protein C25B2.10 | 154.451 | 11 | 1.28833 | 0.00435 |
| Gene.21258::TRINITY_DN18452_c1_g19::g.21258::m.21258 | Putative transferase CAF17, mitochondrial | 285.419 | 14 | 0.71674 | 0.02726 |
| Gene.21285::TRINITY_DN18452_c1_g6::g.21285::m.21285 | T-complex protein 1 subunit gamma | 838.18 | 66 | 0.79856 | 0.00228 |
| Gene.21303::TRINITY_DN18454_c1_g12::g.21303::m.21303 | Respiration factor 2 | 112.079 | 3 | 1.20066 | 0.00773 |
| Gene.21613::TRINITY_DN18467_c1_g2::g.21613::m.21613 | T-complex protein 1 subunit zeta | 764.607 | 40 | 0.81239 | 0.02256 |
| Gene.21665::TRINITY_DN18469_c0_g1::g.21665::m.21665 | Inosine triphosphate pyrophosphatase | 300.056 | 47 | 0.82396 | 0.01163 |
| Gene.21700::TRINITY_DN18471_c0_g13::g.21700::m.21700 | Probable phospholipase C20G8.02, mitochondrial | 283.878 | 2 | 1.30376 | 0.01962 |
| Gene.21723::TRINITY_DN18471_c0_g6::g.21723::m.21723 | DNA-binding protein HEXBP | 147.517 | 75 | 0.79964 | 0.02204 |
| Gene.2177::TRINITY_DN11902_c0_g1::g.2177::m.2177 | Cytochrome P450 monooxygenase, sdnE | 188.734 | 4 | 1.53443 | 0.0398 |
| Gene.21904::TRINITY_DN18478_c1_g6::g.21904::m.21904 | UPF0588 membrane protein C20F10.02c | 161.77 | 10 | 1.24841 | 0.00166 |
| Gene.21921::TRINITY_DN1847_c0_g1::g.21921::m.21921 | Alanyl-tRNA editing protein Aarsd1 | 51.2174 | 31 | 0.66833 | 0.04298 |
| Gene.21940::TRINITY_DN18480_c1_g2::g.21940::m.21940 | Aldehyde reductase 2 | 246.899 | 10 | 1.39577 | 0.03069 |
| Gene.2206::TRINITY_DN11913_c0_g2::g.2206::m.2206 | Translocator protein homolog | 126.716 | 25 | 1.9975 | 0.00021 |
| Gene.22089::TRINITY_DN18488_c2_g13::g.22089::m.22089 | 13 kDa ribonucleoprotein-associated protein | 181.03 | 13 | 0.64249 | 0.01568 |
| Gene.22151::TRINITY_DN18489_c2_g3::g.22151::m.22151 | Spermidine synthase | 458.759 | 47 | 0.76263 | 0.00178 |
| Gene.22164::TRINITY_DN18489_c2_g8::g.22164::m.22164 | Probable E3 ubiquitin-protein ligase WAVH2 | 56.9954 | 5 | 1.24169 | 0.04086 |
| Gene.22218::TRINITY_DN18490_c1_g9::g.22218::m.22218 | Uncharacterized protein YEL023C | 49.2914 | 1 | 1.65722 | 0.02814 |
| Gene.22411::TRINITY_DN18507_c0_g3::g.22411::m.22411 | Heat shock protein hsp88 | 1060.44 | 66 | 0.80995 | 0.03876 |
| Gene.22454::TRINITY_DN18511_c3_g14::g.22454::m.22454 | 60S acidic ribosomal protein P1 | 130.183 | 9 | 0.64564 | 0.01167 |
| Gene.22461::TRINITY_DN18511_c3_g2::g.22461::m.22461 | 2-oxoglutarate-Fe(II) type oxidoreductase hxnY | 146.747 | 59 | 1.3996 | 0.03335 |
| Gene.22543::TRINITY_DN18513_c4_g16::g.22543::m.22543 | GrpE protein homolog, mitochondrial | 255.373 | 53 | 0.66556 | 0.042 |

| | | | | | |
|---|---|---|---|---|---|
| Gene.22682::TRINITY_DN18517_c1_g9::g.22682::m.22682 | Zinc-type alcohol dehydrogenase-like protein C16A3.02c | 199.134 | 28 | 0.7994 | 0.02592 |
| Gene.22745::TRINITY_DN18523_c0_g3::g.22745::m.22745 | N14-3-3 family protein artA | 291.197 | 73 | 0.83262 | 0.02938 |
| Gene.22753::TRINITY_DN18523_c0_g7::g.22753::m.22753 | Reticulocyte-binding protein 2 homolog a | 50.447 | 1 | 2.23276 | 0.04862 |
| Gene.22838::TRINITY_DN18529_c2_g2::g.22838::m.22838 | Dothistromin biosynthesis peroxidase, dotB | 228.794 | 3 | 1.43112 | 0.0294 |
| Gene.22975::TRINITY_DN18534_c1_g5::g.22975::m.22975 | Sphingolipid C9-methyltransferase | 124.405 | 24 | 2.69354 | 0.00643 |
| Gene.23010::TRINITY_DN18535_c0_g16::g.23010::m.23010 | Dihydrofolate reductase | 135.576 | 16 | 0.79021 | 0.0097 |
| Gene.23060::TRINITY_DN18536_c3_g18::g.23060::m.23060 | Mitochondrial import receptor subunit tom70 | 909.057 | 55 | 0.82896 | 0.01604 |
| Gene.23077::TRINITY_DN18536_c3_g27::g.23077::m.23077 | Mannose-6-phosphate isomerase | 235.343 | 7 | 1.75229 | 0.00868 |
| Gene.23123::TRINITY_DN18537_c1_g15::g.23123::m.23123 | U3 small nucleolar RNA-associated protein 5 | 133.265 | 16 | 0.82346 | 0.01725 |
| Gene.23196::TRINITY_DN18539_c0_g14::g.23196::m.23196 | tRNA (guanine-N(7)-)-methyltransferase non-catalytic subunit trm82 | 301.982 | 15 | 0.80102 | 0.00059 |
| Gene.2333::TRINITY_DN11983_c0_g1::g.2333::m.2333 | Replication termination factor 2 | 122.865 | 3 | 0.83008 | 0.04722 |
| Gene.23366::TRINITY_DN18553_c0_g1::g.23366::m.23366 | ATP synthase subunit gamma, mitochondrial | 310.071 | 67 | 0.8218 | 0.00478 |
| Gene.23431::TRINITY_DN18560_c0_g3::g.23431::m.23431 | Glutamine synthetase | 663.685 | 2 | 0.66389 | 0.01378 |
| Gene.2344::TRINITY_DN11985_c0_g1::g.2344::m.2344 | Orotate phosphoribosyltransferase | 283.493 | 47 | 0.81184 | 0.01483 |
| Gene.23467::TRINITY_DN18562_c0_g14::g.23467::m.23467 | Autophagy-related protein 11 | 1475.69 | 1 | 1.28311 | 0.03577 |
| Gene.23498::TRINITY_DN18562_c0_g25::g.23498::m.23498 | Trans-enoyl reductase fsr4 | 314.694 | 38 | 1.56739 | 0.01073 |
| Gene.2358::TRINITY_DN12003_c0_g2::g.2358::m.2358 | Homocysteine synthase | 431.795 | 5 | 1.80767 | 0.03045 |
| Gene.23683::TRINITY_DN18568_c2_g19::g.23683::m.23683 | Probable nitrilase C965.09 | 84.7297 | 2 | 1.80421 | 0.02415 |
| Gene.23766::TRINITY_DN18575_c0_g16::g.23766::m.23766 | TATA element modulatory factor | 81.6481 | 4 | 1.41061 | 0.00182 |
| Gene.23785::TRINITY_DN18575_c0_g25::g.23785::m.23785 | Bifunctional decalin synthase calF | 352.058 | 5 | 1.22552 | 0.02478 |
| Gene.24072::TRINITY_DN18582_c0_g23::g.24072::m.24072 | Laccase-1 | 643.654 | 17 | 2.74703 | 0.00056 |
| Gene.24075::TRINITY_DN18582_c0_g24::g.24075::m.24075 | Laccase-2 | 601.282 | 2 | 1.8436 | 0.00762 |
| Gene.24092::TRINITY_DN18582_c0_g3::g.24092::m.24092 | WSC domain-containing protein ARB_07867 | 849.351 | 32 | 1.8342 | 0.01066 |
| Gene.24116::TRINITY_DN18582_c0_g8::g.24116::m.24116 | Laccase-1 | 646.351 | 10 | 1.6121 | 0.0213 |
| Gene.24133::TRINITY_DN18583_c0_g8::g.24133::m.24133 | Heat shock 70 kDa protein | 731.48 | 56 | 0.6835 | 0.01143 |
| Gene.24177::TRINITY_DN18586_c0_g11::g.24177::m.24177 | Isoleucine--tRNA ligase, chloroplastic / mitochondrial | 641.728 | 18 | 0.76493 | 0.02219 |
| Gene.24250::TRINITY_DN18589_c1_g4::g.24250::m.24250 | Ankyrin-1 | 197.978 | 5 | 0.77778 | 0.00204 |
| Gene.24290::TRINITY_DN18590_c2_g3::g.24290::m.24290 | Glutathione peroxidase-like peroxiredoxin 2 | 202.986 | 22 | 1.36913 | 0.00407 |
| Gene.24391::TRINITY_DN18592_c2_g16::g.24391::m.24391 | NAD(P) transhydrogenase, mitochondrial | 1055.05 | 4 | 1.46812 | 0.02912 |
| Gene.24446::TRINITY_DN18593_c0_g2::g.24446::m.24446 | Mitochondrial protein import protein mas5 | 319.701 | 43 | 0.82039 | 0.02619 |
| Gene.2445::TRINITY_DN12062_c0_g2::g.2445::m.2445 | 25-hydroxycholesterol 7-alpha-hydroxylase (Fragment) | 93.2041 | 6 | 1.30808 | 0.0331 |
| Gene.2466::TRINITY_DN1206_c0_g1::g.2466::m.2466 | Tripeptidyl-peptidase sed1 | 586.645 | 4 | 1.35756 | 0.02477 |
| Gene.24668::TRINITY_DN18601_c1_g16::g.24668::m.24668 | C2H2 finger domain transcription factor crzA | 400.979 | 3 | 0.6802 | 0.0198 |
| Gene.24704::TRINITY_DN18602_c0_g14::g.24704::m.24704 | Serine/threonine-protein kinase RIM15 | 412.535 | 1 | 1.83019 | 0.02033 |
| Gene.24777::TRINITY_DN18603_c0_g14::g.24777::m.24777 | Pyruvate dehydrogenase complex protein X component, mitochondrial | 154.451 | 51 | 0.74723 | 0.00432 |
| Gene.24803::TRINITY_DN18603_c0_g23::g.24803::m.24803 | Galactose/lactose metabolism regulatory protein GAL80 | 126.716 | 29 | 1.36967 | 0.01193 |
| Gene.24841::TRINITY_DN18604_c0_g9::g.24841::m.24841 | 5-methylcytosine rRNA methyltransferase NSUN4 | 83.9593 | 5 | 0.78625 | 0.0333 |
| Gene.2487::TRINITY_DN12084_c0_g3::g.2487::m.2487 | Elongator complex protein 4 | 146.747 | 3 | 1.55754 | 0.00378 |
| Gene.24879::TRINITY_DN18607_c1_g19::g.24879::m.24879 | Probable beta-glucosidase I | 939.488 | 8 | 1.38663 | 0.00154 |
| Gene.24887::TRINITY_DN18607_c1_g21::g.24887::m.24887 | U3 small nucleolar RNA-associated protein 20 | 440.269 | 3 | 1.29797 | 0.00929 |
| Gene.24918::TRINITY_DN18607_c1_g6::g.24918::m.24918 | GBH/ACA ribonucleoprotein complex subunit GAR1 | 194.897 | 20 | 0.66945 | 0.01667 |
| Gene.24941::TRINITY_DN18608_c1_g7::g.24941::m.24941 | Serine/threonine-protein kinase ppk16 | 329.331 | 3 | 1.33827 | 0.01081 |

| | | | | | |
|---|---|---|---|---|---|
| Gene.25250::TRINITY_DN18617_c1_g22::g.25250::m.25250 | Uncharacterized HTH La-type RNA-binding protein C1527.03 | 83.9593 | 26 | 0.70746 | 0.01231 |
| Gene.25255::TRINITY_DN18617_c1_g27::g.25255::m.25255 | Uncharacterized protein C736.07c | 47.3654 | 3 | 1.20994 | 0.02903 |
| Gene.25297::TRINITY_DN18619_c0_g1::g.25297::m.25297 | Ubiquitin-conjugating enzyme E2-16 kDa | 295.049 | 26 | 0.81928 | 0.0047 |
| Gene.25429::TRINITY_DN18623_c2_g16::g.25429::m.25429 | Nucleolar GTP-binding protein 2 | 776.163 | 9 | 0.83262 | 0.02578 |
| Gene.25440::TRINITY_DN18623_c2_g18::g.25440::m.25440 | 40S ribosomal protein S13 | 242.276 | 30 | 1.57137 | 0.00257 |
| Gene.25444::TRINITY_DN18623_c2_g2::g.25444::m.25444 | Anucleate primary sterigmata protein A | 427.172 | 7 | 1.20389 | 0.00173 |
| Gene.25529::TRINITY_DN18625_c0_g14::g.25529::m.25529 | CUE domain-containing protein C354.10 | 78.9518 | 18 | 0.74448 | 0.01152 |
| Gene.25608::TRINITY_DN18626_c1_g4::g.25608::m.25608 | NPC intracellular cholesterol transporter 1-related protein 1 | 867.455 | 9 | 1.22506 | 0.00701 |
| Gene.25652::TRINITY_DN18627_c0_g19::g.25652::m.25652 | Aldehyde dehydrogenase | 393.66 | 2 | 1.29232 | 0.00682 |
| Gene.25657::TRINITY_DN18627_c0_g20::g.25657::m.25657 | Short-chain dehydrogenase/reductase ATR7 | 111.309 | 81 | 1.23343 | 0.0151 |
| Gene.25721::TRINITY_DN18628_c3_g5::g.25721::m.25721 | Probable Delta (7)-sterol 5(6)-desaturase | 503.056 | 3 | 1.51403 | 0.02284 |
| Gene.25910::TRINITY_DN18633_c3_g16::g.25910::m.25910 | Deoxyribonuclease Tat-D | 254.988 | 2 | 0.77567 | 0.01057 |
| Gene.26051::TRINITY_DN18638_c0_g14::g.26051::m.26051 | Putative serine/threonine-protein phosphatase C22H10.04 | 460.299 | 4 | 0.78678 | 0.00489 |
| Gene.2617::TRINITY_DN12157_c0_g1::g.2617::m.2617 | Aspartyl aminopeptidase | 702.205 | 30 | 0.83293 | 0.00038 |
| Gene.26220::TRINITY_DN18643_c1_g11::g.26220::m.26220 | ATP synthase subunit H, mitochondrial | 54.6842 | 36 | 0.76939 | 0.02042 |
| Gene.26310::TRINITY_DN18646_c2_g8::g.26310::m.26310 | Transcription factor SFP1 | 152.525 | 4 | 0.77989 | 0.00317 |
| Gene.26317::TRINITY_DN18646_c2_g9::g.26317::m.26317 | Probable inactive nicotinamidase At3g16190 | 46.2098 | 22 | 1.32648 | 0.00123 |
| Gene.26454::TRINITY_DN18654_c0_g17::g.26454::m.26454 | Small glutamine-rich tetratricopeptide repeat-containing protein 2 | 207.994 | 25 | 0.79051 | 0.00444 |
| Gene.26667::TRINITY_DN18664_c0_g1::g.26667::m.26667 | Ferric/cupric reductase transmembrane component 7 | 139.428 | 11 | 1.3175 | 0.01799 |
| Gene.26690::TRINITY_DN18664_c0_g19::g.26690::m.26690 | Non-specific lipid-transfer protein (Fragment) | 448.358 | 9 | 1.32648 | 0.02921 |
| Gene.26769::TRINITY_DN18665_c1_g6::g.26769::m.26769 | Altered inheritance of mitochondria protein 6 homolog ARB_06966 | 331.643 | 19 | 1.51783 | 0.03495 |
| Gene.27015::TRINITY_DN18671_c6_g7::g.27015::m.27015 | Probable oxygen-dependent coproporphyrinogen-III oxidase | 327.405 | 56 | 0.7336 | 0.03894 |
| Gene.27281::TRINITY_DN18679_c2_g21::g.27281::m.27281 | LETM1 domain-containing protein mdm28, mitochondrial | 385.185 | 40 | 0.79563 | 0.02625 |
| Gene.27399::TRINITY_DN18683_c5_g17::g.27399::m.27399 | Cutinase transcription factor 1 alpha | 76.2554 | 4 | 0.76678 | 0.02396 |
| Gene.27431::TRINITY_DN18683_c5_g6::g.27431::m.27431 | Morphogenesis-related protein MSB1 | 143.665 | 5 | 1.24346 | 0.01609 |
| Gene.2753::TRINITY_DN12228_c0_g1::g.2753::m.2753 | Signaling mucin HKR1 | 56.9954 | 1 | 1.96247 | 0.01314 |
| Gene.27548::TRINITY_DN18693_c0_g1::g.27548::m.27548 | Ketoisovalerate reductase BEA2 | 353.214 | 4 | 1.2193 | 0.01313 |
| Gene.27586::TRINITY_DN18705_c0_g1::g.27586::m.27586 | N-carbamoylputrescine amidase | 82.8037 | 21 | 2.0633 | 0.02486 |
| Gene.27759::TRINITY_DN18778_c0_g1::g.27759::m.27759 | Mitochondrial import inner membrane translocase subunit tim16 | 216.083 | 70 | 0.66759 | 0.03458 |
| Gene.27817::TRINITY_DN18800_c0_g1::g.27817::m.27817 | Probable trafficking protein particle complex subunit 2 | 126.331 | 18 | 2.861 | 0.00945 |
| Gene.27872::TRINITY_DN18827_c0_g1::g.27872::m.27872 | 2-methoxy-6-polyprenyl-1,4-benzoquinol methylase, mitochondrial | 283.108 | 34 | 0.7341 | 0.00594 |
| Gene.27994::TRINITY_DN18897_c0_g1::g.27994::m.27994 | Acid phosphatase PHO1 | 154.451 | 8 | 1.2363 | 0.01129 |
| Gene.28064::TRINITY_DN1893_c0_g1::g.28064::m.28064 | Mitochondrial respiratory chain complexes assembly protein | 764.607 | 45 | 0.77725 | 0.02599 |
| Gene.28174::TRINITY_DN19015_c0_g1::g.28174::m.28174 | Short-chain dehydrogenase reductase 3a | 71.633 | 31 | 0.75109 | 0.00331 |
| Gene.28452::TRINITY_DN1928_c0_g1::g.28452::m.28452 | Short chain dehydrogenase atnD | 196.052 | 47 | 1.23881 | 0.03664 |
| Gene.28634::TRINITY_DN19377_c0_g1::g.28634::m.28634 | ATP-dependent RNA helicase MSS116, mitochondrial | 352.058 | 27 | 0.81623 | 0.00082 |
| Gene.28670::TRINITY_DN19389_c0_g1::g.28670::m.28670 | ATP-dependent RNA helicase mrh4, mitochondrial | 729.554 | 31 | 0.76783 | 0.02727 |
| Gene.28724::TRINITY_DN19415_c0_g1::g.28724::m.28724 | Protein priA | 70.4774 | 5 | 2.88666 | 0.03666 |
| Gene.28736::TRINITY_DN19425_c0_g1::g.28736::m.28736 | Alpha N-terminal protein methyltransferase 1 | 163.31 | 3 | 1.35386 | 0.00255 |
| Gene.28744::TRINITY_DN19432_c0_g1::g.28744::m.28744 | Pre-mRNA-splicing factor SYF1 | 1459.51 | 10 | 1.27704 | 0.03184 |
| Gene.28818::TRINITY_DN19467_c0_g1::g.28818::m.28818 | Plasma alpha-L-fucosidase | 198.749 | 3 | 1.43251 | 0.04929 |
| Gene.28821::TRINITY_DN19469_c0_g1::g.28821::m.28821 | Oxidase FUB9 | 305.834 | 17 | 1.26843 | 0.02801 |

| | | | | | |
|---|---|---|---|---|---|
| Gene.28834::TRINITY_DN19476_c0_g1::g.28834::m.28834 | Protein FMP25, mitochondrial | 120.553 | 41 | 0.80723 | 0.02333 |
| Gene.28914::TRINITY_DN19516_c0_g1::g.28914::m.28914 | 6-phosphogluconate dehydrogenase, decarboxylating | 396.356 | 9 | 1.26453 | 0.03721 |
| Gene.28917::TRINITY_DN19521_c0_g1::g.28917::m.28917 | Succinate-semialdehyde dehydrogenase, mitochondrial | 412.92 | 2 | 0.79319 | 0.00544 |
| Gene.28955::TRINITY_DN19545_c0_g1::g.28955::m.28955 | Hydroxyacyl-thioester dehydratase type 2, mitochondrial | 82.4185 | 13 | 1.31354 | 0.01068 |
| Gene.2900::TRINITY_DN12282_c0_g3::g.2900::m.2900 | Protein BNI4 | 83.5741 | 3 | 1.46324 | 0.00362 |
| Gene.29072::TRINITY_DN19650_c0_g1::g.29072::m.29072 | NADH-ubiquinone oxidoreductase 12 kDa subunit, mitochondrial | 178.718 | 42 | 1.45139 | 0.00809 |
| Gene.29175::TRINITY_DN1977_c0_g1::g.29175::m.29175 | Probable nitrilase C965.09 | 74.3294 | 33 | 1.31392 | 0.03147 |
| Gene.29243::TRINITY_DN1990_c0_g1::g.29243::m.29243 | DnaJ homolog subfamily C member 7 homolog | 325.865 | 31 | 0.73561 | 0.02797 |
| Gene.29258::TRINITY_DN19938_c0_g1::g.29258::m.29258 | Ubiquitin-conjugating enzyme E2 16 | 120.168 | 6 | 1.37758 | 0.01323 |
| Gene.29373::TRINITY_DN20024_c0_g1::g.29373::m.29373 | Nudix hydrolase 7 | 41.9726 | 42 | 0.7991 | 0.02455 |
| Gene.29396::TRINITY_DN20036_c0_g1::g.29396::m.29396 | Neutral ceramidase 2 | 501.516 | 4 | 1.2351 | 0.02762 |
| Gene.29467::TRINITY_DN2006_c0_g1::g.29467::m.29467 | UPF0768 protein YBL029C-A | 39.6614 | 6 | 0.67673 | 0.03301 |
| Gene.29625::TRINITY_DN20133_c0_g1::g.29625::m.29625 | Ras-like protein | 385.185 | 70 | 1.30238 | 0.03152 |
| Gene.29694::TRINITY_DN20172_c0_g1::g.29694::m.29694 | Elongator complex protein 2 | 560.066 | 3 | 1.28958 | 0.02862 |
| Gene.2978::TRINITY_DN12352_c0_g1::g.2978::m.2978 | Glycerophosphocholine permease, GIT4 | 259.996 | 11 | 1.75735 | 0.00396 |
| Gene.29832::TRINITY_DN20250_c0_g1::g.29832::m.29832 | 54S ribosomal protein L28, mitochondrial | 42.743 | 14 | 0.58228 | 0.01726 |
| Gene.29848::TRINITY_DN20259_c0_g1::g.29848::m.29848 | Tryprostatin B 6-hydroxylase | 316.62 | 4 | 1.65913 | 0.02441 |
| Gene.29876::TRINITY_DN20288_c0_g1::g.29876::m.29876 | Putative agmatinase 1 | 533.487 | 19 | 1.58398 | 0.00681 |
| Gene.29987::TRINITY_DN20384_c0_g1::g.29987::m.29987 | Imidazoleglycerol-phosphate dehydratase | 398.667 | 36 | 0.75622 | 0.0034 |
| Gene.30027::TRINITY_DN20432_c0_g1::g.30027::m.30027 | Probable proteasome subunit alpha type-6 | 315.849 | 56 | 0.8226 | 0.00154 |
| Gene.30062::TRINITY_DN20461_c0_g1::g.30062::m.30062 | 54S ribosomal protein L19, mitochondrial | 157.918 | 38 | 0.80451 | 0.04828 |
| Gene.30115::TRINITY_DN20520_c0_g1::g.30115::m.30115 | Monoogygenase CPUR_05431 | 202.601 | 30 | 1.61097 | 0.02665 |
| Gene.30156::TRINITY_DN20574_c0_g1::g.30156::m.30156 | Cytoplasmic tRNA 2-thiolation protein 2 | 120.939 | 14 | 1.28175 | 0.00296 |
| Gene.30185::TRINITY_DN20617_c0_g1::g.30185::m.30185 | Uncharacterized oxidoreductase C663.08c | 114.005 | 6 | 1.53528 | 0.01423 |
| Gene.30355::TRINITY_DN20867_c0_g1::g.30355::m.30355 | Chromatin structure-remodeling complex subunit, sfh1 | 141.739 | 4 | 1.33463 | 0.03126 |
| Gene.30439::TRINITY_DN20899_c0_g1::g.30439::m.30439 | Phospholipid-transporting ATPase DNF1 | 1413.67 | 6 | 1.28311 | 0.01049 |
| Gene.30587::TRINITY_DN20943_c0_g1::g.30587::m.30587 | 54S ribosomal protein L36, mitochondrial | 55.0694 | 20 | 0.79802 | 0.01928 |
| Gene.30618::TRINITY_DN20955_c0_g1::g.30618::m.30618 | Mitochondrial zinc maintenance protein 1, mitochondrial | 129.413 | 28 | 0.72534 | 0.00514 |
| Gene.30640::TRINITY_DN20963_c0_g1::g.30640::m.30640 | Uncharacterized protein C23C11.06c | 270.396 | 9 | 1.47975 | 0.03452 |
| Gene.30648::TRINITY_DN20966_c0_g1::g.30648::m.30648 | Uncharacterized protein C32A11.02c | 310.071 | 29 | 1.54453 | 0.01059 |
| Gene.30686::TRINITY_DN20986_c0_g1::g.30686::m.30686 | Uncharacterized protein C16G5.07c | 372.474 | 59 | 0.75872 | 0.03873 |
| Gene.30763::TRINITY_DN21028_c0_g1::g.30763::m.30763 | FAD-linked oxidoreductase ZEB1 | 531.946 | 4 | 1.85987 | 0.04909 |
| Gene.30836::TRINITY_DN21073_c0_g1::g.30836::m.30836 | Calcium channel YVC1 | 450.284 | 6 | 1.25188 | 0.01931 |
| Gene.30845::TRINITY_DN21077_c0_g1::g.30845::m.30845 | Acetyl-coenzyme A transferase nodX | 196.823 | 18 | 1.54669 | 0.00857 |
| Gene.30971::TRINITY_DN21143_c0_g1::g.30971::m.30971 | Pepsin B | 43.8986 | 2 | 1.7688 | 0.01787 |
| Gene.31032::TRINITY_DN21177_c0_g1::g.31032::m.31032 | Vacuolar membrane protease | 995.342 | 10 | 1.42188 | 0.00296 |
| Gene.31097::TRINITY_DN21233_c0_g1::g.31097::m.31097 | Mitochondrial import inner membrane translocase subunit tim44 | 327.02 | 53 | 0.7336 | 0.02742 |
| Gene.31334::TRINITY_DN21478_c0_g1::g.31334::m.31334 | 40S ribosomal protein S11-A | 221.861 | 16 | 4.28546 | 0.01486 |
| Gene.31570::TRINITY_DN21748_c0_g1::g.31570::m.31570 | Putative NADPH dehydrogenase C23G7.10c | 309.301 | 2 | 1.38135 | 0.0324 |
| Gene.31578::TRINITY_DN21751_c0_g1::g.31578::m.31578 | Solute carrier family 25 member 40 | 233.417 | 3 | 0.79319 | 0.00465 |
| Gene.31676::TRINITY_DN21789_c0_g1::g.31676::m.31676 | Probable secreted lipase ARB_00047 | 212.616 | 3 | 1.75149 | 0.00321 |
| Gene.31752::TRINITY_DN21824_c0_g1::g.31752::m.31752 | Translation initiation factor IF-2, mitochondrial | 489.96 | 12 | 0.82648 | 0.02754 |

| | | | | | |
|---|---|---|---|---|---|
| Gene.31881::TRINITY_DN21893_c0_g1::g.31881::m.31881 | Amino-acid permease 2 | 586.645 | 3 | 1.63968 | 0.03315 |
| Gene.31888::TRINITY_DN21897_c0_g1::g.31888::m.31888 | Phytoene desaturase | 642.114 | 24 | 1.84091 | 0.00026 |
| Gene.31951::TRINITY_DN21926_c0_g1::g.31951::m.31951 | Mitochondrial carnitine carrier | 273.863 | 23 | 1.34467 | 0.0301 |
| Gene.32028::TRINITY_DN21962_c0_g1::g.32028::m.32028 | 3-dehydroshikimate dehydratase | 150.599 | 9 | 2.96367 | 0.0111 |
| Gene.32091::TRINITY_DN22004_c0_g1::g.32091::m.32091 | Methyltransferase-like protein 7B | 77.7962 | 17 | 1.26548 | 0.02139 |
| Gene.32127::TRINITY_DN22021_c0_g1::g.32127::m.32127 | Probable 54S ribosomal protein L32, mitochondrial | 77.0258 | 7 | 1.71985 | 0.02186 |
| Gene.32132::TRINITY_DN22024_c0_g1::g.32132::m.32132 | Pre-mRNA-splicing factor sap61 | 416.387 | 14 | 1.29008 | 0.00052 |
| Gene.32242::TRINITY_DN22121_c0_g1::g.32242::m.32242 | Imidazole glycerol phosphate synthase hisHF | 803.127 | 30 | 0.78253 | 0.01417 |
| Gene.32261::TRINITY_DN22133_c0_g1::g.32261::m.32261 | Uncharacterized protein tam6, mitochondrial | 43.8986 | 6 | 0.82871 | 0.02084 |
| Gene.32263::TRINITY_DN22137_c0_g1::g.32263::m.32263 | Glycerol-3-phosphate dehydrogenase [NAD(+)] | 639.032 | 18 | 0.82588 | 0.00354 |
| Gene.32332::TRINITY_DN22203_c0_g1::g.32332::m.32332 | Iron-sulfur assembly protein 2 | 80.8777 | 8 | 0.72662 | 0.0301 |
| Gene.32520::TRINITY_DN22558_c0_g1::g.32520::m.32520 | Uncharacterized protein C1840.09 | 183.341 | 41 | 0.7316 | 0.02021 |
| Gene.32548::TRINITY_DN22567_c0_g1::g.32548::m.32548 | Vacuolar morphogenesis protein 7 homolog | 127.102 | 22 | 1.33775 | 0.03618 |
| Gene.32551::TRINITY_DN22569_c0_g1::g.32551::m.32551 | Protein BFR2 | 431.409 | 7 | 1.40152 | 0.00592 |
| Gene.32564::TRINITY_DN22578_c0_g1::g.32564::m.32564 | Cytochrome c oxidase polypeptide 5, mitochondrial | 114.775 | 11 | 2.15126 | 0.0185 |
| Gene.32600::TRINITY_DN22595_c0_g1::g.32600::m.32600 | Probable lysosomal cobalamin transporter | 808.135 | 5 | 1.23214 | 0.01591 |
| Gene.32614::TRINITY_DN22599_c0_g1::g.32614::m.32614 | Uncharacterized CDP-alcohol phosphatidyltransferase class-I family protein C22A12.08c | 239.965 | 6 | 1.59516 | 0.02438 |
| Gene.32678::TRINITY_DN22626_c0_g1::g.32678::m.32678 | Lysine--tRNA ligase, mitochondrial | 308.531 | 15 | 0.79319 | 0.00868 |
| Gene.3268::TRINITY_DN12468_c0_g1::g.3268::m.3268 | Retrograde regulation protein 2 | 231.106 | 5 | 1.2531 | 0.01833 |
| Gene.32764::TRINITY_DN22658_c0_g1::g.32764::m.32764 | Putative D-lactate dehydrogenase C713.03, mitochondrial | 637.876 | 34 | 0.81214 | 0.03628 |
| Gene.32848::TRINITY_DN22702_c0_g1::g.32848::m.32848 | Mitochondrial amidoxime-reducing component 1 | 72.4034 | 3 | 1.3706 | 0.03866 |
| Gene.32888::TRINITY_DN22717_c0_g1::g.32888::m.32888 | Vacuolar transporter chaperone 3 | 455.292 | 28 | 1.3175 | 0.01562 |
| Gene.32898::TRINITY_DN22719_c0_g1::g.32898::m.32898 | AFG1-like ATPase | 142.124 | 4 | 1.28002 | 0.02697 |
| Gene.32918::TRINITY_DN22726_c0_g1::g.32918::m.32918 | eEF1A lysine and N-terminal methyltransferase | 58.5362 | 3 | 1.27407 | 0.00222 |
| Gene.33030::TRINITY_DN22773_c0_g1::g.33030::m.33030 | Cytochrome P450 monooxygenase paxP | 145.591 | 4 | 1.73179 | 0.02279 |
| Gene.33036::TRINITY_DN22777_c0_g1::g.33036::m.33036 | Copper amine oxidase 1 | 444.121 | 3 | 0.73611 | 0.00166 |
| Gene.33220::TRINITY_DN22865_c0_g1::g.33220::m.33220 | Mitochondrial import inner membrane translocase subunit TIM50 | 603.208 | 34 | 0.73992 | 0.04134 |
| Gene.33361::TRINITY_DN22949_c0_g1::g.33361::m.33361 | 54S ribosomal protein L17, mitochondrial | 127.102 | 29 | 0.7508 | 0.01443 |
| Gene.33380::TRINITY_DN22965_c0_g1::g.33380::m.33380 | GTPase-activating protein GYP5 | 257.684 | 1 | 0.66113 | 0.01532 |
| Gene.33429::TRINITY_DN229_c0_g1::g.33429::m.33429 | Putative copper transporter crmD | 43.8986 | 5 | 1.52419 | 0.04004 |
| Gene.33434::TRINITY_DN23007_c0_g1::g.33434::m.33434 | Microfibrillar-associated protein 1 | 112.849 | 2 | 1.57689 | 0.01842 |
| Gene.33451::TRINITY_DN23016_c0_g1::g.33451::m.33451 | 54S ribosomal protein L11, mitochondrial | 64.3142 | 34 | 0.76574 | 0.00504 |
| Gene.33483::TRINITY_DN23034_c0_g1::g.33483::m.33483 | HD domain-containing protein C4G3.17 | 141.739 | 22 | 1.26548 | 0.02747 |
| Gene.33551::TRINITY_DN23102_c0_g1::g.33551::m.33551 | Probable cytosolic iron-sulfur protein assembly protein 1 | 530.02 | 5 | 0.80505 | 0.00494 |
| Gene.33576::TRINITY_DN2311_c0_g2::g.33576::m.33576 | Agroclavine dehydrogenase | 234.187 | 5 | 1.26672 | 0.04222 |
| Gene.33630::TRINITY_DN23182_c0_g1::g.33630::m.33630 | 3-hydroxyacyl-CoA dehydrogenase type-2 | 56.9954 | 26 | 1.49273 | 0.02049 |
| Gene.33667::TRINITY_DN2322_c0_g1::g.33667::m.33667 | FAD-dependent monooxygenase sorC | 180.644 | 8 | 1.76243 | 0.04917 |
| Gene.33762::TRINITY_DN23337_c0_g1::g.33762::m.33762 | Putative redox protein fmp46, mitochondrial | 56.225 | 53 | 0.706 | 0.0366 |
| Gene.33861::TRINITY_DN2367_c0_g1::g.33861::m.33861 | Uncharacterized RNA-binding protein C1861.04c | 298.13 | 14 | 1.41198 | 0.00796 |
| Gene.33873::TRINITY_DN2371_c0_g1::g.33873::m.33873 | U3 small nucleolar RNA-associated protein 15 | 345.51 | 6 | 0.72989 | 0.02025 |
| Gene.33901::TRINITY_DN2396_c0_g1::g.33901::m.33901 | Probable vacuolar protease A | 90.8929 | 8 | 1.28746 | 0.03967 |

| | | | | | |
|---|---|---|---|---|---|
| Gene.33920::TRINITY_DN2411_c0_g1::g.33920::m.33920 | Mitochondrial inner membrane protein OXA1L | 108.612 | 16 | 0.77883 | 0.01179 |
| Gene.33985::TRINITY_DN2457_c0_g1::g.33985::m.33985 | Syntaxin-22 | 103.219 | 26 | 1.27311 | 0.00591 |
| Gene.34003::TRINITY_DN2480_c0_g1::g.34003::m.34003 | BSD domain-containing protein C22A12.14c | 85.8853 | 39 | 0.77433 | 0.00108 |
| Gene.34287::TRINITY_DN2699_c0_g1::g.34287::m.34287 | Short chain dehydrogenase mpl6 | 143.28 | 46 | 2.38792 | 0.00029 |
| Gene.3431::TRINITY_DN12520_c0_g1::g.3431::m.3431 | Translation initiation factor eIF-2B subunit gamma | 120.553 | 15 | 0.81159 | 0.03743 |
| Gene.34314::TRINITY_DN2716_c0_g1::g.34314::m.34314 | Citrinin biosynthesis transcriptional activator ctnR | 165.236 | 1 | 1.2363 | 0.01511 |
| Gene.3444::TRINITY_DN12530_c0_g3::g.3444::m.3444 | Dihydropyrimidinase | 399.053 | 22 | 1.48139 | 0.01219 |
| Gene.34497::TRINITY_DN2901_c0_g1::g.34497::m.34497 | Trafficking protein particle complex subunit 11 | 77.7962 | 2 | 0.77567 | 0.03019 |
| Gene.34536::TRINITY_DN295_c0_g1::g.34536::m.34536 | Protein Rf1, mitochondrial | 70.8626 | 1 | 0.82704 | 0.00389 |
| Gene.34606::TRINITY_DN3026_c0_g1::g.34606::m.34606 | 5-aminolevulinate synthase, erythroid-specific, mitochondrial | 68.5514 | 7 | 1.44698 | 0.00095 |
| Gene.34730::TRINITY_DN3158_c0_g1::g.34730::m.34730 | Cytochrome P450 monooxygenase sdnH | 465.692 | 9 | 2.05499 | 0.01929 |
| Gene.34777::TRINITY_DN31_c0_g1::g.34777::m.34777 | Dehydrogenase patE | 555.058 | 31 | 1.74303 | 0.00458 |
| Gene.34928::TRINITY_DN3372_c0_g1::g.34928::m.34928 | Homoserine dehydrogenase | 411.764 | 75 | 0.8105 | 0.01057 |
| Gene.35013::TRINITY_DN3498_c0_g1::g.35013::m.35013 | Zerumbone synthase | 145.976 | 18 | 1.50522 | 0.01882 |
| Gene.35141::TRINITY_DN3598_c0_g2::g.35141::m.35141 | Elongation factor G, mitochondrial | 1267.68 | 39 | 0.78808 | 0.00711 |
| Gene.35235::TRINITY_DN3683_c0_g1::g.35235::m.35235 | RNA-capping enzyme subunit alpha | 255.758 | 4 | 1.20426 | 0.04758 |
| Gene.35243::TRINITY_DN368_c0_g1::g.35243::m.35243 | DNA-directed RNA polymerases I, II, and III subunit RPABC2 | 148.288 | 5 | 0.65062 | 0.01932 |
| Gene.35256::TRINITY_DN3693_c0_g1::g.35256::m.35256 | Outer spore wall protein RRT8 | 43.8986 | 3 | 1.55102 | 0.02058 |
| Gene.35323::TRINITY_DN3764_c0_g1::g.35323::m.35323 | Ubiquinone biosynthesis protein, coq9 | 134.42 | 12 | 0.75007 | 0.02403 |
| Gene.35461::TRINITY_DN38_c0_g1::g.35461::m.35461 | AB hydrolase superfamily protein C4A8.06c | 57.3806 | 11 | 1.35849 | 0.01832 |
| Gene.356::TRINITY_DN10547_c0_g1::g.356::m.356 | Mitochondrial phosphate carrier protein 2 | 236.113 | 45 | 1.53915 | 0.03053 |
| Gene.35605::TRINITY_DN4020_c0_g1::g.35605::m.35605 | GPN-loop GTPase 1 | 409.068 | 14 | 0.73561 | 0.01049 |
| Gene.35609::TRINITY_DN4020_c0_g2::g.35609::m.35609 | DNA-directed RNA polymerases I, II, and III subunit RPABC4 | 77.411 | 21 | 0.69895 | 0.00207 |
| Gene.35664::TRINITY_DN406_c0_g1::g.35664::m.35664 | Glucose-6-phosphate isomerase | 149.828 | 6 | 1.79367 | 0.03087 |
| Gene.35806::TRINITY_DN4185_c0_g1::g.35806::m.35806 | Aromatic amino acid aminotransferase C56E4.03 | 251.521 | 11 | 1.24551 | 0.00253 |
| Gene.35818::TRINITY_DN4197_c0_g1::g.35818::m.35818 | Probable exocyst complex component sec8 | 1415.59 | 9 | 1.23297 | 0.01422 |
| Gene.35928::TRINITY_DN429_c0_g1::g.35928::m.35928 | Uncharacterized leucine-rich repeat-containing protein C926.06c | 243.432 | 1 | 1.40577 | 0.00903 |
| Gene.35936::TRINITY_DN4303_c0_g1::g.35936::m.35936 | Sexual differentiation process protein isp4 | 467.618 | 1 | 1.8851 | 0.02691 |
| Gene.35941::TRINITY_DN4305_c0_g1::g.35941::m.35941 | Iron-sulfur assembly protein 1 | 141.739 | 4 | 0.71478 | 5.7E-05 |
| Gene.36053::TRINITY_DN4357_c0_g1::g.36053::m.36053 | Serine/threonine-protein kinase KIN28 | 290.426 | 4 | 0.81573 | 0.02647 |
| Gene.36240::TRINITY_DN4442_c0_g1::g.36240::m.36240 | Uncharacterized protein C3B8.09 | 239.195 | 4 | 1.48798 | 0.03177 |
| Gene.3639::TRINITY_DN12608_c0_g3::g.3639::m.3639 | Exosome complex component CSL4 | 155.992 | 19 | 1.2805 | 0.0053 |
| Gene.36401::TRINITY_DN450_c0_g2::g.36401::m.36401 | Replication factor A protein 3 | 67.781 | 35 | 0.76753 | 0.01444 |
| Gene.36449::TRINITY_DN4526_c0_g1::g.36449::m.36449 | Dedicator of cytokinesis protein 1 | 338.576 | 3 | 1.35942 | 0.00126 |
| Gene.36456::TRINITY_DN4529_c0_g1::g.36456::m.36456 | DNA mismatch repair protein msh-2 | 1655.19 | 2 | 1.90508 | 0.046 |
| Gene.36494::TRINITY_DN4550_c0_g1::g.36494::m.36494 | Probable proteasome subunit alpha type-2 | 474.167 | 36 | 0.82785 | 0.00662 |
| Gene.36611::TRINITY_DN4610_c0_g1::g.36611::m.36611 | Septum-promoting GTP-binding protein 1 | 348.977 | 18 | 1.6455 | 0.00568 |
| Gene.36616::TRINITY_DN4611_c0_g1::g.36616::m.36616 | Trafficking protein particle complex subunit BET3 | 222.246 | 12 | 0.76419 | 0.00107 |
| Gene.36618::TRINITY_DN4612_c0_g1::g.36618::m.36618 | Uncharacterized bolA-like protein C4B3.11c | 84.7297 | 6 | 0.79994 | 0.01144 |
| Gene.36693::TRINITY_DN4636_c0_g2::g.36693::m.36693 | Polyadenylation factor subunit 2 | 753.821 | 3 | 1.35756 | 0.0022 |
| Gene.36763::TRINITY_DN4656_c0_g2::g.36763::m.36763 | tRNA-specific adenosine deaminase subunit tad3 | 81.2629 | 5 | 1.41935 | 0.03064 |
| Gene.36832::TRINITY_DN4684_c0_g1::g.36832::m.36832 | Inner nuclear membrane protein SRC1 | 89.3521 | 18 | 1.22058 | 0.00592 |

| | | | | | |
|---|---|---|---|---|---|
| Gene.36922::TRINITY_DN4728_c0_g2::g.36922::m.36922 | Phosphatidylinositol 4-phosphate 5-kinase its3 | 559.681 | 3 | 0.7549 | 0.02121 |
| Gene.36929::TRINITY_DN4729_c0_g1::g.36929::m.36929 | Transforming growth factor-beta receptor-associated protein 1 | 100.523 | 1 | 1.28137 | 0.01534 |
| Gene.36942::TRINITY_DN4733_c0_g1::g.36942::m.36942 | Vacuolar transporter chaperone 1 | 175.252 | 15 | 1.21685 | 0.0421 |
| Gene.36984::TRINITY_DN4756_c0_g1::g.36984::m.36984 | SEH-associated protein 4 | 223.402 | 2 | 1.34703 | 0.00795 |
| Gene.37026::TRINITY_DN4780_c0_g1::g.37026::m.37026 | Lariat debranching enzyme | 335.109 | 2 | 1.20588 | 0.02347 |
| Gene.37064::TRINITY_DN4810_c0_g1::g.37064::m.37064 | Uncharacterized methyltransferase C70.08c | 70.4774 | 49 | 1.41643 | 0.03485 |
| Gene.3726::TRINITY_DN12682_c0_g2::g.3726::m.3726 | ADIPOR-like receptor IZH2 | 165.236 | 3 | 0.8018 | 0.00096 |
| Gene.37269::TRINITY_DN5092_c0_g1::g.37269::m.37269 | Ribose-phosphate pyrophosphokinase 5 | 389.808 | 20 | 1.21031 | 0.00194 |
| Gene.3744::TRINITY_DN12690_c0_g1::g.3744::m.3744 | WW domain-containing oxidoreductase | 56.9954 | 18 | 1.45943 | 0.01585 |
| Gene.37487::TRINITY_DN5320_c0_g1::g.37487::m.37487 | WHI2-like protein P4H10.16c | 162.925 | 26 | 1.44458 | 0.00266 |
| Gene.37530::TRINITY_DN5351_c0_g1::g.37530::m.37530 | Cytochrome P450 52A13 | 296.204 | 14 | 1.51889 | 0.02507 |
| Gene.37726::TRINITY_DN5593_c0_g2::g.37726::m.37726 | Dehydrodolichyl diphosphate synthase complex subunit NUS1 | 176.022 | 8 | 1.21685 | 0.01111 |
| Gene.37948::TRINITY_DN5805_c0_g2::g.37948::m.37948 | Uncharacterized protein C9E9.15 | 115.931 | 9 | 0.71009 | 0.0249 |
| Gene.37993::TRINITY_DN5827_c0_g1::g.37993::m.37993 | Phosphatidylglycerol phospholipase C | 113.235 | 16 | 0.67832 | 0.01385 |
| Gene.3810::TRINITY_DN12739_c0_g2::g.3810::m.3810 | Uncharacterized protein YLL056C | 63.929 | 45 | 1.33372 | 0.02965 |
| Gene.38197::TRINITY_DN596_c0_g1::g.38197::m.38197 | Phenylalanine--tRNA ligase, mitochondrial | 272.322 | 29 | 0.73611 | 0.00942 |
| Gene.38273::TRINITY_DN6048_c0_g1::g.38273::m.38273 | 26S proteasome non-ATPase regulatory subunit 9 | 141.739 | 16 | 0.80807 | 0.03237 |
| Gene.38307::TRINITY_DN6085_c0_g1::g.38307::m.38307 | All-trans-retinol dehydrogenase [NAD(+)] ADH1B | 151.754 | 34 | 1.24972 | 0.01105 |
| Gene.38764::TRINITY_DN6515_c0_g2::g.38764::m.38764 | Acetate permease A | 279.256 | 9 | 1.7894 | 0.03858 |
| Gene.3881::TRINITY_DN12771_c0_g1::g.3881::m.3881 | V-type proton ATPase 16 kDa proteolipid subunit | 260.766 | 12 | 1.46264 | 0.0005 |
| Gene.38843::TRINITY_DN6555_c0_g1::g.38843::m.38843 | Kynureninase 2 | 666.381 | 4 | 0.75879 | 0.00466 |
| Gene.39008::TRINITY_DN6665_c0_g2::g.39008::m.39008 | Solute carrier family 28 member 3 | 281.182 | 2 | 1.46163 | 0.04352 |
| Gene.3904::TRINITY_DN12783_c0_g3::g.3904::m.3904 | Putative arrestin-related trafficking adapter C2D10.04 | 265.774 | 4 | 1.32558 | 0.00275 |
| Gene.39087::TRINITY_DN6726_c0_g1::g.39087::m.39087 | Putative mitochondrial carnitine O-acetyltransferase | 516.924 | 8 | 1.31622 | 0.01688 |
| Gene.39179::TRINITY_DN67_c0_g1::g.39179::m.39179 | Coactosin | 106.686 | 42 | 0.82315 | 0.00414 |
| Gene.3927::TRINITY_DN12792_c0_g1::g.3927::m.3927 | Mitochondrial import inner membrane translocase subunit tim22 | 234.958 | 24 | 0.41878 | 0.01354 |
| Gene.39288::TRINITY_DN6902_c0_g1::g.39288::m.39288 | Putative nucleosome assembly protein C36B7.08c | 85.1149 | 20 | 0.82562 | 0.0462 |
| Gene.39374::TRINITY_DN7016_c0_g1::g.39374::m.39374 | Acetyl-CoA--deacetylcephalosporin C acetyltransferase | 61.6178 | 7 | 1.39561 | 0.00839 |
| Gene.3939::TRINITY_DN1279_c0_g2::g.3939::m.3939 | Acetamidase | 165.622 | 17 | 1.25649 | 0.02072 |
| Gene.39422::TRINITY_DN7076_c0_g1::g.39422::m.39422 | ATP synthase subunit epsilon, mitochondrial | 44.669 | 22 | 0.81984 | 0.02458 |
| Gene.39539::TRINITY_DN717_c0_g1::g.39539::m.39539 | Sphingosine N-acyltransferase-like protein FUM18 | 358.607 | 1 | 1.25602 | 0.03607 |
| Gene.39616::TRINITY_DN7212_c0_g1::g.39616::m.39616 | Choline monooxygenase, chloroplastic | 130.568 | 2 | 1.8342 | 0.03263 |
| Gene.39624::TRINITY_DN7218_c0_g3::g.39624::m.39624 | Carboxyvinyl-carboxyphosphonate phosphorylmutase, chloroplastic | 157.918 | 19 | 1.29747 | 0.00833 |
| Gene.39704::TRINITY_DN7262_c0_g1::g.39704::m.39704 | Protein SIP5 | 499.975 | 6 | 1.27752 | 0.01059 |
| Gene.39760::TRINITY_DN7290_c0_g1::g.39760::m.39760 | Mitochondrial ATPase complex subunit ATP10 | 97.4413 | 19 | 0.72068 | 0.02554 |
| Gene.39769::TRINITY_DN7299_c0_g1::g.39769::m.39769 | Trans-enoyl reductase fsdC | 132.109 | 3 | 1.38434 | 0.01649 |
| Gene.39801::TRINITY_DN7350_c0_g2::g.39801::m.39801 | Acetyl-CoA acetyltransferase, mitochondrial | 172.94 | 5 | 0.72911 | 0.03328 |
| Gene.39819::TRINITY_DN7374_c0_g2::g.39819::m.39819 | Alpha-L-arabinofuranosidase C | 583.948 | 3 | 2.30943 | 0.04115 |
| Gene.39901::TRINITY_DN7465_c0_g2::g.39901::m.39901 | pH-response regulator protein palA/RIM20 | 1119.76 | 10 | 1.20913 | 0.00073 |
| Gene.39907::TRINITY_DN747_c0_g1::g.39907::m.39907 | Cytochrome P450 monooxygenase aclL | 370.163 | 3 | 1.47219 | 0.01285 |
| Gene.40007::TRINITY_DN75_c0_g1::g.40007::m.40007 | Uncharacterized membrane protein C24H6.13 | 395.586 | 3 | 1.21848 | 0.02898 |
| Gene.40045::TRINITY_DN7629_c0_g1::g.40045::m.40045 | Uncharacterized membrane protein YJR124C | 218.779 | 10 | 1.55168 | 0.01086 |

| | | | | | |
|---|---|---|---|---|---|
| Gene.40282::TRINITY_DN7893_c1_g1::g.40282::m.40282 | Probable U6 snRNA-associated Sm-like protein LSm3 | 110.538 | 14 | 0.80397 | 0.01806 |
| Gene.40317::TRINITY_DN7917_c0_g2::g.40317::m.40317 | Pre-rRNA-processing protein rix1 | 45.0542 | 10 | 0.8276 | 0.02956 |
| Gene.40435::TRINITY_DN8040_c0_g1::g.40435::m.40435 | Short-chain dehydrogenase/reductase tropE | 274.633 | 34 | 1.9513 | 0.00662 |
| Gene.40471::TRINITY_DN8072_c0_g1::g.40471::m.40471 | NADH dehydrogenase [ubiquinone] iron-sulfur protein 5-B | 55.0694 | 44 | 1.57843 | 0.04246 |
| Gene.40536::TRINITY_DN8105_c0_g1::g.40536::m.40536 | tRNA-dihydrouridine(20) synthase [NAD(P)+] | 244.973 | 4 | 1.21402 | 0.00079 |
| Gene.40547::TRINITY_DN8111_c0_g1::g.40547::m.40547 | NO-methyltransferase cnsE | 99.3673 | 9 | 1.24972 | 0.03908 |
| Gene.40636::TRINITY_DN8146_c0_g1::g.40636::m.40636 | Dolichol kinase sec59 | 141.739 | 1 | 1.25442 | 0.01945 |
| Gene.40734::TRINITY_DN8212_c0_g1::g.40734::m.40734 | Microtubule integrity protein mal3 | 227.254 | 60 | 0.73489 | 0.00921 |
| Gene.40760::TRINITY_DN8225_c0_g1::g.40760::m.40760 | Subtilisin-like proteinase Spm1 | 589.726 | 30 | 1.20345 | 0.00331 |
| Gene.40854::TRINITY_DN8256_c0_g1::g.40854::m.40854 | Putative lipase YOR059C | 51.6026 | 6 | 1.94551 | 0.00886 |
| Gene.41031::TRINITY_DN8315_c0_g2::g.41031::m.41031 | Carboxypeptidase S1 homolog A | 593.964 | 7 | 1.4818 | 0.01807 |
| Gene.4115::TRINITY_DN12879_c0_g5::g.4115::m.4115 | Phytanoyl-CoA dioxygenase domain-containing protein 1 homolog | 213.386 | 21 | 1.29446 | 0.00012 |
| Gene.4122::TRINITY_DN12882_c0_g1::g.4122::m.4122 | 10 kDa heat shock protein, mitochondrial | 128.257 | 11 | 0.62646 | 0.04336 |
| Gene.41268::TRINITY_DN8412_c0_g2::g.41268::m.41268 | Bax inhibitor 1 | 179.489 | 5 | 1.36274 | 0.01528 |
| Gene.4136::TRINITY_DN12891_c0_g1::g.4136::m.4136 | Aurofusarin biosynthesis cluster protein S | 141.739 | 24 | 1.457 | 0.04935 |
| Gene.41424::TRINITY_DN8547_c0_g1::g.41424::m.41424 | Translation machinery-associated protein 46 | 267.314 | 22 | 0.83293 | 0.02048 |
| Gene.41452::TRINITY_DN8568_c0_g1::g.41452::m.41452 | Probable very-long-chain (3R)-3-hydroxyacyl-CoA dehydratase | 145.206 | 3 | 1.39672 | 0.03577 |
| Gene.41478::TRINITY_DN8579_c0_g1::g.41478::m.41478 | AP-3 complex subunit beta-2 | 418.698 | 7 | 1.36794 | 0.00072 |
| Gene.41483::TRINITY_DN8580_c0_g1::g.41483::m.41483 | Copper amine oxidase 1 | 823.543 | 21 | 1.27187 | 0.00281 |
| Gene.41514::TRINITY_DN8604_c0_g2::g.41514::m.41514 | Acyl-CoA synthetase FUM16 | 712.22 | 12 | 1.26159 | 0.00916 |
| Gene.41602::TRINITY_DN8663_c0_g1::g.41602::m.41602 | 26S proteasome regulatory subunit rpn11 | 455.292 | 38 | 0.72414 | 0.00118 |
| Gene.41644::TRINITY_DN8688_c0_g2::g.41644::m.41644 | DNA-directed RNA polymerase III subunit rpc9 | 54.6842 | 19 | 0.80204 | 0.02398 |
| Gene.41810::TRINITY_DN8831_c0_g1::g.41810::m.41810 | Catalase-1 | 1064.29 | 26 | 1.45399 | 0.02659 |
| Gene.4184::TRINITY_DN12913_c0_g2::g.4184::m.4184 | Acyl-CoA dehydrogenase AFT10-1 | 356.681 | 12 | 1.34558 | 0.03338 |
| Gene.41848::TRINITY_DN88_c0_g1::g.41848::m.41848 | DnaJ homolog 1, mitochondrial | 498.049 | 24 | 0.65472 | 0.01413 |
| Gene.41898::TRINITY_DN8960_c0_g1::g.41898::m.41898 | Methionine aminopeptidase 1 | 447.203 | 10 | 1.23501 | 0.01382 |
| Gene.41997::TRINITY_DN905_c0_g1::g.41997::m.41997 | 2-aminomuconic semialdehyde dehydrogenase | 425.246 | 7 | 1.42482 | 0.02231 |
| Gene.42051::TRINITY_DN9119_c0_g1::g.42051::m.42051 | Protein CASP | 252.292 | 15 | 1.24551 | 0.00299 |
| Gene.42102::TRINITY_DN9158_c0_g1::g.42102::m.42102 | Probable transcriptional regulator ycf27 | 85.1149 | 66 | 2.67586 | 0.04451 |
| Gene.42188::TRINITY_DN9248_c0_g1::g.42188::m.42188 | Methylenetetrahydrofolate dehydrogenase [NAD(+)] | 375.17 | 38 | 0.78731 | 0.04149 |
| Gene.42189::TRINITY_DN9248_c0_g2::g.42189::m.42189 | Signal peptidase complex subunit SPC2 | 69.707 | 17 | 1.21484 | 0.0085 |
| Gene.42379::TRINITY_DN9404_c1_g2::g.42379::m.42379 | Methyltransferase trt5 | 84.7297 | 20 | 1.25056 | 0.02366 |
| Gene.42769::TRINITY_DN9682_c0_g2::g.42769::m.42769 | Bifunctional lycopene cyclase/phytoene synthase | 548.51 | 16 | 1.41198 | 0.01422 |
| Gene.42789::TRINITY_DN9695_c0_g1::g.42789::m.42789 | ABC multidrug transporter mdr1 | 1712.2 | 11 | 2.33148 | 9.1E-06 |
| Gene.42884::TRINITY_DN9762_c0_g2::g.42884::m.42884 | HD domain-containing protein C4G3.17 | 172.555 | 28 | 0.81354 | 0.01495 |
| Gene.42922::TRINITY_DN9783_c0_g1::g.42922::m.42922 | Iron-sulfur clusters transporter ATM1, mitochondrial | 387.497 | 7 | 1.21529 | 0.02467 |
| Gene.42927::TRINITY_DN9789_c0_g1::g.42927::m.42927 | Adrenodoxin homolog, mitochondrial | 168.318 | 6 | 0.68473 | 0.04773 |
| Gene.42943::TRINITY_DN9792_c0_g1::g.42943::m.42943 | Hydrolase tropI | 192.971 | 32 | 1.25649 | 0.00151 |
| Gene.4298::TRINITY_DN12969_c0_g1::g.4298::m.4298 | ATP-dependent RNA helicase DBP8 | 776.548 | 7 | 1.38379 | 0.01127 |
| Gene.43004::TRINITY_DN9842_c0_g1::g.43004::m.43004 | Protein sum2 | 132.88 | 13 | 0.77335 | 0.0049 |
| Gene.43085::TRINITY_DN9944_c0_g1::g.43085::m.43085 | Tyrosine--tRNA ligase, cytoplasmic | 160.229 | 7 | 0.81184 | 0.04814 |
| Gene.4324::TRINITY_DN12984_c0_g1::g.4324::m.4324 | Tubulin-specific chaperone D | 70.8626 | 8 | 1.28871 | 0.01281 |

| | | | | | |
|---|---|---|---|---|---|
| Gene.4349::TRINITY_DN12996_c0_g2::g.4349::m.4349 | Uncharacterized oxidoreductase C162.03 | 157.147 | 5 | 1.37021 | 0.01715 |
| Gene.4455::TRINITY_DN13039_c0_g2::g.4455::m.4455 | Probable NADH dehydrogenase | 304.294 | 14 | 0.69827 | 0.01245 |
| Gene.4581::TRINITY_DN13079_c0_g3::g.4581::m.4581 | Meiotically up-regulated gene 190 protein | 806.979 | 3 | 1.27101 | 0.01762 |
| Gene.4649::TRINITY_DN13109_c0_g2::g.4649::m.4649 | Cytochrome c oxidase assembly protein COX20, mitochondrial | 73.559 | 17 | 0.73461 | 0.00416 |
| Gene.4665::TRINITY_DN13117_c0_g6::g.4665::m.4665 | Monooxygenase CTB7 | 196.052 | 20 | 2.44037 | 0.01564 |
| Gene.4868::TRINITY_DN13182_c0_g2::g.4868::m.4868 | Mitochondrial presequence protease | 1541.17 | 44 | 0.74672 | 0.0194 |
| Gene.4965::TRINITY_DN13228_c0_g1::g.4965::m.4965 | Probable T-complex protein 1 subunit eta | 831.247 | 44 | 0.83094 | 0.03208 |
| Gene.5140::TRINITY_DN13416_c0_g1::g.5140::m.5140 | 30S ribosomal protein S12, chloroplastic | 204.912 | 28 | 2.12175 | 0.00542 |
| Gene.5260::TRINITY_DN13571_c0_g2::g.5260::m.5260 | Sphingolipid C4-hydroxylase SUR2 | 271.166 | 12 | 1.22222 | 0.00693 |
| Gene.5288::TRINITY_DN13606_c0_g2::g.5288::m.5288 | Monoogygenase | 241.891 | 23 | 1.68456 | 0.01026 |
| Gene.5452::TRINITY_DN13766_c0_g2::g.5452::m.5452 | Mitochondrial-processing peptidase subunit alpha | 694.501 | 37 | 0.78366 | 0.00091 |
| Gene.55::TRINITY_DN10100_c0_g1::g.55::m.55 | U3 small nucleolar ribonucleoprotein protein IMP3 | 141.739 | 10 | 0.77485 | 0.01533 |
| Gene.5579::TRINITY_DN1390_c0_g1::g.5579::m.5579 | Putative amidase ARB_02965 | 537.339 | 4 | 1.3482 | 0.03155 |
| Gene.571::TRINITY_DN10722_c1_g1::g.571::m.571 | Putative amidase C869.01 | 619.387 | 3 | 1.365 | 0.01406 |
| Gene.5752::TRINITY_DN14117_c0_g1::g.5752::m.5752 | Alpha-1,2-mannosyltransferase alg9 | 376.326 | 5 | 1.76927 | 0.02433 |
| Gene.5807::TRINITY_DN14158_c0_g3::g.5807::m.5807 | Altered inheritance of mitochondria protein 18, mitochondrial | 119.398 | 35 | 0.78018 | 0.00356 |
| Gene.5842::TRINITY_DN14178_c1_g2::g.5842::m.5842 | Vacuolar protein-sorting-associated protein 11 homolog | 444.121 | 3 | 1.23214 | 0.00232 |
| Gene.5887::TRINITY_DN14221_c0_g1::g.5887::m.5887 | 2,3-bisphosphoglycerate-independent phosphoglycerate mutase | 548.51 | 61 | 0.79611 | 0.01204 |
| Gene.5958::TRINITY_DN14264_c0_g4::g.5958::m.5958 | UBX domain-containing protein 1 | 245.358 | 35 | 0.80156 | 0.0165 |
| Gene.6376::TRINITY_DN14570_c0_g3::g.6376::m.6376 | Lon protease homolog, mitochondrial | 1607.81 | 39 | 0.7092 | 0.0051 |
| Gene.6401::TRINITY_DN14592_c0_g2::g.6401::m.6401 | Peroxiredoxin | 269.24 | 34 | 0.69539 | 0.0088 |
| Gene.6534::TRINITY_DN14669_c0_g1::g.6534::m.6534 | Mannan polymerase II complex ANP1 subunit | 484.182 | 6 | 1.30858 | 0.02079 |
| Gene.6555::TRINITY_DN14688_c0_g2::g.6555::m.6555 | Exosome complex component rrp45 | 312.383 | 17 | 0.75 | 0.01021 |
| Gene.6610::TRINITY_DN14705_c0_g6::g.6610::m.6610 | Zinc-type alcohol dehydrogenase-like protein C16A3.02c | 176.407 | 21 | 1.51298 | 0.01543 |
| Gene.6752::TRINITY_DN14798_c0_g2::g.6752::m.6752 | Nuclear polyadenylated RNA-binding protein NAB2 | 72.0182 | 7 | 0.80096 | 0.00476 |
| Gene.6806::TRINITY_DN14838_c0_g2::g.6806::m.6806 | Acyl-CoA dehydrogenase family member 9, mitochondrial | 48.1358 | 17 | 1.28484 | 0.02829 |
| Gene.6807::TRINITY_DN14840_c1_g1::g.6807::m.6807 | Probable quinone oxidoreductase | 229.565 | 2 | 0.81269 | 0.0371 |
| Gene.683::TRINITY_DN10799_c0_g2::g.683::m.683 | Short-chain dehydrogenase/reductase ATR7 | 80.4925 | 70 | 0.74672 | 0.01411 |
| Gene.6863::TRINITY_DN14869_c0_g4::g.6863::m.6863 | Tyrosinase ustQ | 131.724 | 12 | 1.34611 | 0.0009 |
| Gene.6949::TRINITY_DN14942_c0_g1::g.6949::m.6949 | Histone acetyltransferase ESA1 | 770 | 3 | 1.23048 | 0.0074 |
| Gene.6995::TRINITY_DN1496_c0_g1::g.6995::m.6995 | Ribokinase | 195.282 | 21 | 0.75901 | 0.00103 |
| Gene.7044::TRINITY_DN15000_c0_g1::g.7044::m.7044 | Uricase | 373.244 | 40 | 1.57333 | 0.0009 |
| Gene.7130::TRINITY_DN1505_c0_g1::g.7130::m.7130 | Proline utilization trans-activator | 55.0694 | 1 | 1.72773 | 0.04687 |
| Gene.7292::TRINITY_DN15158_c0_g2::g.7292::m.7292 | re-mRNA-splicing factor cwf19 | 400.593 | 2 | 1.32108 | 0.00065 |
| Gene.7383::TRINITY_DN15207_c0_g1::g.7383::m.7383 | DOA4-independent degradation protein 4 | 200.29 | 15 | 1.22717 | 0.00808 |
| Gene.7421::TRINITY_DN15226_c0_g2::g.7421::m.7421 | Transcriptional regulatory protein dep1 | 73.559 | 3 | 0.71625 | 0.03347 |
| Gene.7453::TRINITY_DN1524_c0_g1::g.7453::m.7453 | Oxidoreductase andH | 149.443 | 16 | 1.33502 | 0.0311 |
| Gene.7509::TRINITY_DN15280_c0_g2::g.7509::m.7509 | O-acetyltransferase ptmV | 110.923 | 2 | 1.69618 | 0.02163 |
| Gene.7575::TRINITY_DN15315_c0_g2::g.7575::m.7575 | Probable aminotransferase aclI | 449.514 | 2 | 1.23214 | 0.00928 |
| Gene.7593::TRINITY_DN15322_c0_g1::g.7593::m.7593 | SAGA-associated factor 29 | 102.834 | 3 | 1.24383 | 0.01895 |
| Gene.7599::TRINITY_DN15326_c0_g1::g.7599::m.7599 | Thioredoxin reductase gliT | 279.641 | 33 | 1.45659 | 0.03178 |
| Gene.763::TRINITY_DN10857_c0_g2::g.763::m.763 | t-SNARE affecting a late Golgi compartment protein 1 | 56.9954 | 15 | 0.74927 | 0.01364 |

| | | | | | |
|---|---|---|---|---|---|
| Gene.7630::TRINITY_DN15343_c0_g1::g.7630::m.7630 | Uncharacterized transcriptional regulatory protein C3C7.04 | 129.798 | 2 | 1.32468 | 0.01331 |
| Gene.7833::TRINITY_DN15451_c0_g4::g.7833::m.7833 | Putative nitroreductase HBN1 | 42.743 | 34 | 0.77021 | 0.01314 |
| Gene.7887::TRINITY_DN15476_c0_g2::g.7887::m.7887 | Failed axon connections | 75.0998 | 27 | 0.78412 | 0.00537 |
| Gene.7910::TRINITY_DN15485_c0_g1::g.7910::m.7910 | Bifunctional solanapyrone synthase | 184.882 | 1 | 1.86533 | 0.00296 |
| Gene.7920::TRINITY_DN15494_c0_g1::g.7920::m.7920 | Probable RNA-binding protein 19 | 93.9745 | 53 | 0.75718 | 0.01876 |
| Gene.797::TRINITY_DN10880_c0_g3::g.797::m.797 | Acid phosphatase | 152.525 | 10 | 1.67618 | 0.03717 |
| Gene.8048::TRINITY_DN15572_c0_g1::g.8048::m.8048 | Translation machinery-associated protein 7 | 53.5286 | 20 | 0.59872 | 0.00419 |
| Gene.8237::TRINITY_DN15683_c0_g1::g.8237::m.8237 | Uncharacterized transcriptional regulatory protein C1327.01c | 60.8474 | 1 | 1.52674 | 0.04518 |
| Gene.8367::TRINITY_DN15727_c0_g2::g.8367::m.8367 | Trimethyllysine dioxygenase | 494.197 | 11 | 1.29008 | 0.00744 |
| Gene.8371::TRINITY_DN15728_c0_g1::g.8371::m.8371 | Phosphatidylglycerol phospholipase C | 134.806 | 8 | 1.59024 | 0.01076 |
| Gene.838::TRINITY_DN10913_c0_g1::g.838::m.838 | Ribosomal RNA-processing protein 14 | 90.8929 | 2 | 0.72237 | 0.00876 |
| Gene.8380::TRINITY_DN15733_c1_g1::g.8380::m.8380 | Probable D-xylulose reductase A | 120.168 | 34 | 0.81628 | 0.00551 |
| Gene.8557::TRINITY_DN15826_c0_g3::g.8557::m.8557 | 4-trimethylaminobutyraldehyde dehydrogenase | 164.466 | 22 | 1.39848 | 0.03785 |
| Gene.8690::TRINITY_DN1590_c0_g1::g.8690::m.8690 | Proline iminopeptidase PfmaB | 541.576 | 9 | 1.23131 | 0.00397 |
| Gene.8743::TRINITY_DN15947_c0_g1::g.8743::m.8743 | T-complex protein 1 subunit delta | 742.265 | 49 | 0.80668 | 0.00732 |
| Gene.8799::TRINITY_DN15972_c0_g1::g.8799::m.8799 | Dihydrolipoyllysine-residue acetyltransferase component of pyruvate dehydrogenase complex, mitochondrial | 615.15 | 53 | 0.72761 | 0.00221 |
| Gene.880::TRINITY_DN10955_c0_g1::g.880::m.880 | Uncharacterized protein C56F8.02 | 1506.12 | 9 | 1.20147 | 0.00179 |
| Gene.8809::TRINITY_DN15975_c0_g1::g.8809::m.8809 | Probable U6 snRNA-associated Sm-like protein LSm4 | 143.665 | 41 | 0.79503 | 0.00594 |
| Gene.8893::TRINITY_DN1601_c0_g1::g.8893::m.8893 | Ribosome biogenesis protein nsa2 | 451.825 | 2 | 0.59251 | 0.00332 |
| Gene.8981::TRINITY_DN16076_c0_g1::g.8981::m.8981 | Probable T-complex protein 1 subunit beta | 791.956 | 59 | 0.80699 | 0.00363 |
| Gene.9102::TRINITY_DN16125_c0_g3::g.9102::m.9102 | ABC multidrug transporter atrF | 1181.39 | 3 | 1.36967 | 0.03644 |
| Gene.9137::TRINITY_DN16132_c0_g2::g.9137::m.9137 | Proteasome subunit alpha type-4 | 343.969 | 63 | 0.79235 | 0.00021 |
| Gene.9235::TRINITY_DN16158_c0_g1::g.9235::m.9235 | Nuclear cap-binding protein subunit 2 | 192.971 | 8 | 0.73511 | 0.00365 |
| Gene.9273::TRINITY_DN16171_c0_g1::g.9273::m.9273 | Phosphate-repressible acid phosphatase | 292.352 | 5 | 1.45861 | 0.04885 |
| Gene.9811::TRINITY_DN16322_c0_g1::g.9811::m.9811 | 15-cis-phytoene desaturase, chloroplastic/chromoplastic | 52.7582 | 19 | 1.21113 | 0.0251 |
| Gene.9826::TRINITY_DN16324_c0_g1::g.9826::m.9826 | Uncharacterized protein YGR266W | 397.127 | 2 | 1.21857 | 0.01486 |
| Gene.9894::TRINITY_DN16342_c0_g1::g.9894::m.9894 | Tat-binding homolog 7 | 671.389 | 5 | 1.2531 | 0.03254 |
| Gene.9906::TRINITY_DN16346_c0_g1::g.9906::m.9906 | Mitochondrial import inner membrane translocase subunit tim23 | 195.282 | 28 | 0.73461 | 0.01813 |
| Gene.9944::TRINITY_DN16356_c0_g1::g.9944::m.9944 | Nucleoprotein TPR | 236.113 | 15 | 1.24346 | 0.02617 |
| Gene.9988::TRINITY_DN16365_c0_g5::g.9988::m.9988 | Itaconate transport protein | 271.937 | 4 | 2.90371 | 0.02744 |
